# Tissue-specific transcriptome profiling of the Arabidopsis inflorescence stem reveals local cellular signatures

**DOI:** 10.1093/plcell/koaa019

**Published:** 2020-12-02

**Authors:** Dongbo Shi, Virginie Jouannet, Javier Agustí, Verena Kaul, Victor Levitsky, Pablo Sanchez, Victoria V Mironova, Thomas Greb

**Affiliations:** 1 Department of Developmental Physiology, Centre for Organismal Studies (COS), Heidelberg University, Im Neuenheimer Feld 230, 69120 Heidelberg, Germany; 2 Japan Science and Technology Agency (JST), Saitama, Kawaguchi, Japan; 3 Gregor Mendel Institute (GMI), Austrian Academy of Sciences, Vienna Biocenter (VBC), Dr. Bohr-Gasse 3, 1030 Vienna, Austria; 4 Faculty of Natural Sciences, Novosibirsk State University, Novosibirsk, 630090, Russia; 5 Department of Systems Biology, Institute of Cytology and Genetics, Siberian Branch, Russian Academy of Sciences, Novosibirsk, 630090, Russia; 6 Department of Plant Systems Physiology, Institute for Water and Wetland Research, Radboud University, Heyendaalseweg 135, 6525 AJ Nijmegen, The Netherlands; 7 Instituto de Biología Molecular y Celular de Plantas (IBMCP), Universitat Politècnica de València (UPV)-Consejo Superior de Investigaciones Científicas (CSIC), C/Enginyer Fausto Elio S/N. 46011 Valencia, Spain

## Abstract

Genome-wide gene expression maps with a high spatial resolution have substantially accelerated plant molecular science. However, the number of characterized tissues and growth stages is still small due to the limited accessibility of most tissues for protoplast isolation. Here, we provide gene expression profiles of the mature inflorescence stem of *Arabidopsis thaliana* covering a comprehensive set of distinct tissues. By combining fluorescence-activated nucleus sorting and laser-capture microdissection with next-generation RNA sequencing, we characterized the transcriptomes of xylem vessels, fibers, the proximal and distal cambium, phloem, phloem cap, pith, starch sheath, and epidermis cells. Our analyses classified more than 15,000 genes as being differentially expressed among different stem tissues and revealed known and novel tissue-specific cellular signatures. By determining overrepresented transcription factor binding regions in the promoters of differentially expressed genes, we identified candidate tissue-specific transcriptional regulators. Our datasets predict the expression profiles of an exceptional number of genes and allow hypotheses to be generated about the spatial organization of physiological processes. Moreover, we demonstrate that information about gene expression in a broad range of mature plant tissues can be established at high spatial resolution by nuclear mRNA profiling. Tissue-specific gene expression values can be accessed online at https://arabidopsis-stem.cos.uni-heidelberg.de/.

## Teaser

A genome-wide high-resolution gene expression map of the Arabidopsis inflorescence stem is provided, shedding light on cell type properties and the spatial organization of physiological processes.

## Introduction

Characterizing gene expression in individual cell types is a powerful tool for revealing local molecular signatures in multicellular organisms. By combining genetically encoded fluorescent reporters driven by tissue-specific promoters and fluorescence-activated cell sorting (FACS), high-resolution gene expression profiles have been established for developmental hotspots such as the root and shoot tips of *Arabidopsis thaliana* (Arabidopsis) (Birnbaum et al., 2003; Brady et al., 2007; Yadav et al., 2009, 2014; Wendrich et al., 2020). Although these datasets have served as central resources for the scientific community for many years, high-resolution gene expression maps have not been developed for many other organs or tissues. One of the obstacles is the reliable isolation of RNA from more differentiated tissues and cell types. Depending on their identity or developmental stage, plant cells are surrounded by cell walls with very diverse properties. This requires extensive tuning of the methods used to disrupt cell walls for each case individually (Bart et al., 2006; Lin et al., 2014) or hampers the isolation overall. Even when protoplasts can be isolated, their highly diverse sizes render the subsequent sorting process challenging. Laser capture microdissection (LCM) is an alternative tool for the precise isolation of cellular material (Schad et al., 2005; Chandran et al., 2010; Agusti et al., 2011; Blokhina et al., 2016). The spatial specificity of the profiling process is lower in this case, because genetically encoded markers for cell identity can hardly be used. However, LCM is a powerful method when genetically encoded markers are not available and cell types can be clearly identified by morphology or anatomical position.

**Figure:**
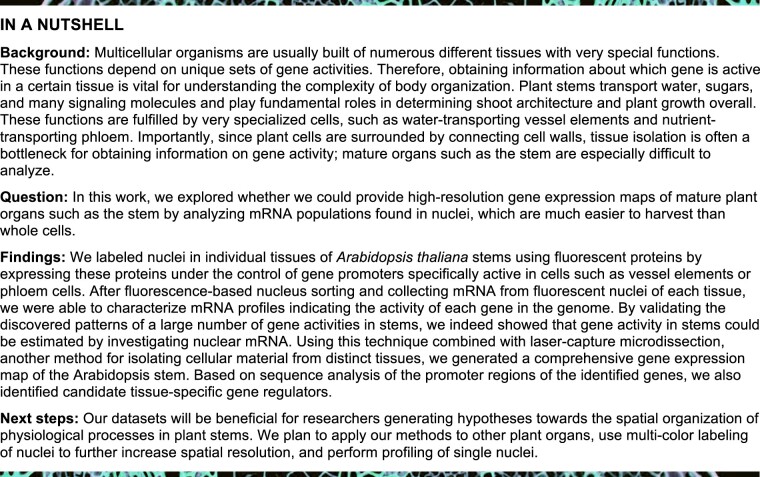


**Figure 1:**
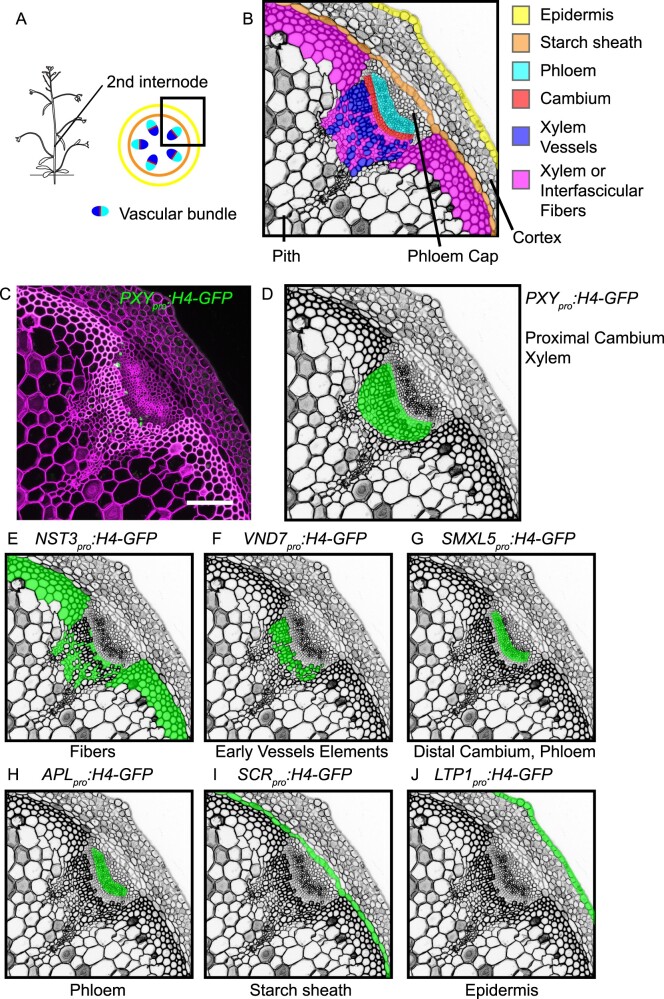
Expression patterns of *H4-GFP* transgenes in the second internode. (A and B) Representations of tissue configuration in the second bottom-most Arabidopsis internode, as seen in cross-sections. The region shown in B corresponds to the black squared region in A. (C) Maximum intensity projection of confocal images of internode sections of the *PXY_pro_:H4-GFP* line. The GFP signal is shown in green, and cell walls are stained with Direct Red 23 and visualized in magenta. Scale bar: 100 µm. Note that only nuclei in the limited observable depth of the section are visualized. (D**–**J) Schematic indication of activity patterns of the different *H4-GFP* transgenes in cross sections of the second internode. Original data are shown in Supplemental Figure 1.

Plant stems play fundamental roles in determining shoot architecture and act as transport routes that connect photosynthetically active source organs with the remaining plant body (Sanchez et al., 2012; Serrano-Mislata and Sablowski, 2018). After the transition from vegetative to reproductive growth, Arabidopsis forms inflorescence stems, which are composed of various tissues including the epidermis, cortex, starch sheath, vascular bundles, and pith (Figure 1, A and B). These tissues fulfill very specialized roles in the plant body and, by acting in concert, form the stem as a functional unit. For example, the epidermis protects the plant from desiccation by building a transpiration barrier and serves as a first line of defense against pathogens (Esau, 1977; Suh et al., 2005). In turn, the starch sheath, also designated as endodermis, executes gravity sensing (Morita et al., 2002; Nawrath et al., 2013). In vascular bundles, xylem and phloem tissues, which are composed of specialized cell types such as xylem fibers, xylem vessel elements, phloem sieve elements, and phloem companion cells, enable water and nutrient transport (Figure 1B). In contrast to these highly specialized cells, cambium stem cells maintain the potential to generate secondary xylem and phloem cells to increase the transport capacity and mechanical support of the growing shoot system (Suer et al., 2011). Interestingly, many of the tissues found in stems are also found in other organs such as roots or leaves. However, the organ-specific profiles of these tissues remain to be characterized systematically.

Arabidopsis stems, like those in most dicotyledonous species, undergo a major anatomical transition during the initiation of radial growth and extensive wood formation (Sanchez et al., 2012). In stems holding a primary tissue conformation, cambium stem cells are restricted to vascular bundles, whereas in secondary stems, cambium cells are also found in interfascicular regions. In this manner, cambium stem cells establish concentric domains of vascular tissues important for organized radial growth (Sehr et al., 2010; Sanchez et al., 2012).

Considering the central role of plant stems in determining plant architecture and physiology, it is vital to have information about gene expression profiles from a comprehensive set of cell types and tissues. From these data, physiological and developmental features of stem tissues can be revealed and the organ can be characterized as a functional unit. Several studies in aspen (*Populus sp.*) have provided high-resolution transcriptional profiles using serial cryo-sectioning (Schrader et al., 2004; Immanen et al., 2016; Sundell et al., 2017), taking advantage of the large organ size of this plant. In Arabidopsis, although several studies have provided transcriptome profiles from several stem tissues and stages (Ko and Han, 2004; Ko et al., 2004; Suh et al., 2005; Brackmann et al., 2018), systematic transcriptome analyses of specific stem tissues at specific stages (such as the pith and the phloem cap) are pending.

Due to the large organ diameter and heterogeneity of cell walls in plant stems, enzymatic protoplast generation appears to be unsuitable for harvesting material from different stem tissues with equal efficiency. Therefore, nucleus isolation represents an attractive alternative. Using this technique, nuclei are released after manual chopping (Galbraith et al., 1983) and can be isolated from individual tissues based on biotin labeling of the nuclear envelope and subsequent immunoprecipitation (Deal and Henikoff, 2010) or fluorescence-activated nucleus sorting (FANS) (Zhang et al., 2005, 2008; Slane et al., 2014; Gutzat et al., 2020). Moreover, although nuclear mRNA and cytosolic mRNA have different compositions and roles (Yang et al., 2017; Choudury et al., 2019), in general, cellular gene expression can be deduced accurately based on nuclear mRNA levels (Zhang et al., 2008; Deal and Henikoff, 2010; You et al., 2017; Palovaara and Weijers, 2019).

Here, motivated by these considerations, we employed FANS and LCM to extract and profile mRNA from a large set of tissues in the primary Arabidopsis stem. By using tissue-specific promoters for fluorescence labeling and profiling nuclei from seven tissues and LCM for two tissues for which no specific promoter was identified, we revealed spatial information about gene activities in a genome-wide fashion. Because the primary inflorescence stem contains a large spectrum of tissues including extremes, such as cambium stem cells and terminally differentiated cells in the vasculature, our results demonstrate the broad applicability of these approaches.

## Results

### Establishment of plant lines for tissue-specific labeling of nuclei

To establish experimental access to mRNA from individual stem tissues, we first screened the literature for promoters that are specifically active in distinct tissues (Schürholz et al., 2018). As a result, we chose the *NAC SECONDARY WALL THICKENING PROMOTING3* (*NST3*) promoter to label fibers (Mitsuda et al., 2007); the *VASCULAR-RELATED NAC DOMAIN PROTEIN7* (*VND7*) promoter for differentiating vessel elements (Kubo et al., 2005; Yamaguchi et al., 2010); the *PHLOEM INTERCALATED WITH XYLEM* (*PXY*)*/TDIF RECEPTOR* (*TDR*) promoter for differentiating xylem cells and proximal cambium cells (Fisher and Turner, 2007; Hirakawa et al., 2008; Shi et al., 2019); the *SMAX1-LIKE5* (*SMXL5*) promoter for differentiating phloem cells and distal cambium cells (Wallner et al., 2017, 2020; Shi et al., 2019); the *ALTERED PHLOEM DEVELOPMENT* (*APL*) promoter for differentiated phloem cells (Bonke et al., 2003; Agustí et al., 2011); the *SCARECROW* (*SCR*) promoter for starch sheath cells (Wysocka-Diller et al., 2000); and the *LIPID TRANSFER PROTEIN1* (*LTP1*) promoter for epidermis cells (Baroux et al., 2001; Figure 1B). To confirm tissue-specificity of promoter activities, we generated transgenic Arabidopsis lines expressing a fusion protein between histone H4 and green fluorescent protein (H4-GFP) under the control of each promoter (*GENE_pro_:H4-GFP*). Microscopic inspection of cross-sections from the second bottom-most elongated internode showed that only nuclei in the expected tissues were labeled by GFP (Figure 1C–J; Supplemental Figure 1), indicating that our lines carried GFP-positive nuclei in a tissue-specific manner.

### Tissue-specific gene activity in stems can be determined by nuclear mRNA profiling

**Figure 2:**
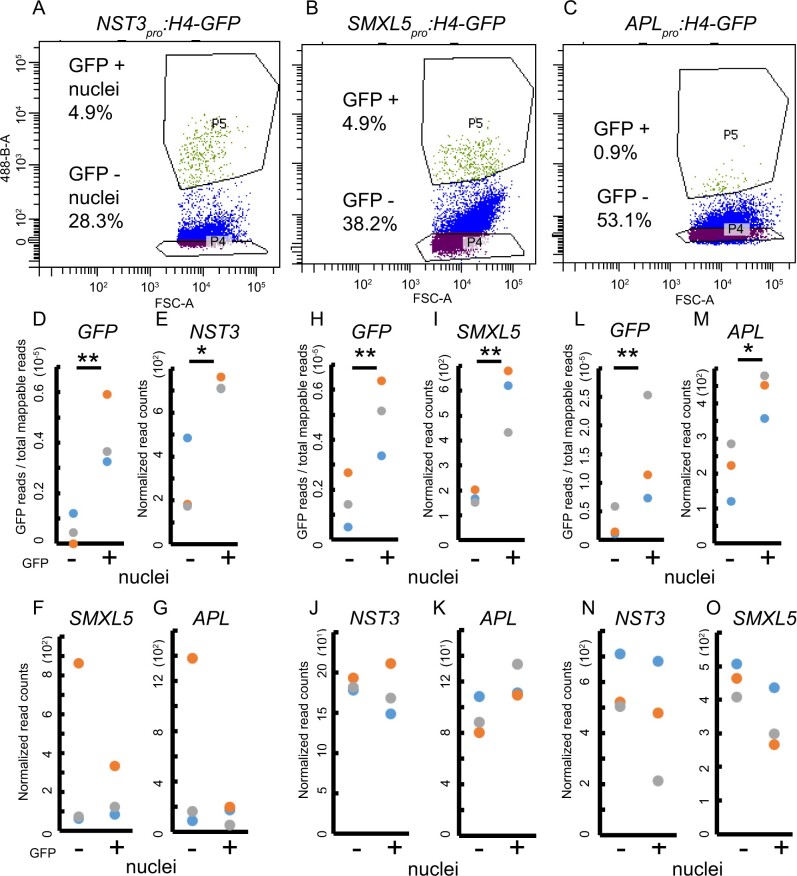
Sorting gates and gene expression analyses of GFP-positive or GFP-negative nuclei. (A**–**C) Plots of gates defining GFP-positive nuclei (P5) and GFP-negative nuclei (P4) while sorting nuclei from the second internode of *NST3_pro_:H4-GFP* (**A**), *SMXL5_pro_:H4-GFP* (B) and *APL_pro_:H4-GFP* (C) lines, respectively. The ratios of each population compared with all particle counts are labeled. The X axis (FSC intensity) indicates the diameter of particles, the Y axis (488) indicates GFP fluorescence. (D**, **H**,** and L) Comparison of read counts mapped to the *GFP* sequence to the total number of mappable reads to the *Arabidopsis* genome in GFP-positive and GFP-negative nuclei for each transgenic line. *n* = 3 for each population for each line. **P < 0.01 in the inverted beta-binomial test. (E–G, I–K, M–O) Normalized read counts for *NST3*, *SMXL5*, and *APL* in GFP-positive and GFP-negative nuclei for *NST3_pro_:H4-GFP* (E–G), *SMXL5_pro_:H4-GFP* (I–K) and *APL_pro_:H4-GFP* (M–O) lines, respectively. *n* = 3 for each population. **P < 0.01 and *P < 0.05 determined by the Wald test.

To determine whether we were able to faithfully extract tissue-specific mRNA, we first focused on inner tissues and tissues producing prominent secondary cell walls, as we expected that mRNA isolation would be most challenging in these cases. Therefore, we collected nuclei from fibers marked by *NST3*_pro_ activity, the distal cambium (*SMXL5*_pro_), and the phloem (*APL*_pro_) by manually chopping the second bottom-most elongated internodes of inflorescence stems, followed by FANS. From each line, we harvested GFP-positive and -negative nuclei (Figure 2A–C; Supplemental Figure 2) and processed 15,000 nuclei per sample for transcriptome analyses (Figure 2A–C) by employing SMART-seq2 amplification of mRNA (Picelli et al., 2013) and RNA-seq analysis of three replicates per sample type. Following this strategy, we found that the levels of the *H4-GFP* mRNA (detected through the *GFP* sequence) and the mRNA of the *NST3*, *SMXL5*, or *APL* genes whose promoters were used to express H4-GFP in the respective lines were significantly higher in extracts from GFP-positive nuclei compared with extracts from GFP-negative nuclei in all cases (Figure 2D–O; *P *<* *0.01 in the inverted beta-binomial test or *P *<* *0.05 in the Wald test). These findings indicate that cell type-specific transcriptomes were thoroughly accessible following our experimental pipeline. To determine the analytical potential of the obtained datasets, we carried out differential expression analysis and found that the transcriptional activity from 23, 40, and 115 genes was significantly higher in GFP-positive nuclei of the *NST3*_pro_, *SMXL5*_pro_, and *APL*_pro_ reporter lines, respectively, compared with GFP-negative nuclei [Supplemental Data Set 1, Benjamini–Hochberg {BH} adjustment of *P* <0.05 in the Wald test]. The relatively low number of tissue-specific genes identified in the one-to-one comparisons was mostly due to the noisy character of datasets from GFP-negative nuclei, which also resulted in a lack of significance for the higher activities of the *NST3* and *APL* genes in the respective tissues after using BH adjustment (Supplemental Data Set 1).

### Transcriptome analysis of seven stem tissues reveals tissue-specific gene activity

**Figure 3:**
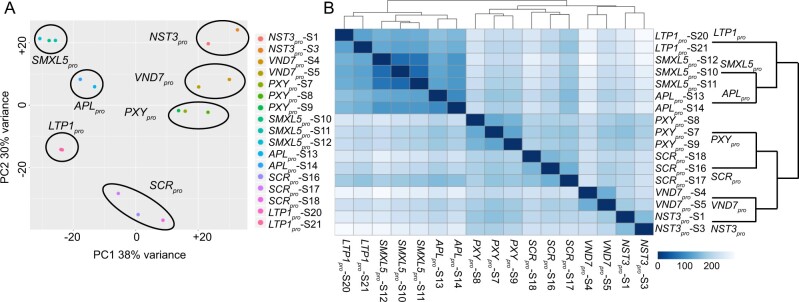
Statistical comparisons of RNA-seq datasets derived from GFP-positive nuclei of seven stem tissues. (A) PCA of log2-transformed normalized read counts of each RNA-seq dataset. (B) Heatmap displaying the statistical distance between each RNA-seq dataset according to the color code. Euclidean distance [arbitrary unit] between samples was calculated using the *dist* function in R, based on the log2-transformed normalized read counts. Two or three replicates were obtained from each nucleus population. The simplified dendrogram to the right of the heatmap represents the hierarchical relationship between the different tissue types.

To address this point, we performed transcriptome profiling of GFP-positive nuclei harvested from the four remaining lines (*PXY*_pro_, *LTP1*_pro_, *VND7*_pro_, *SCR*_pro_; Supplemental Figure 2A–D) by RNA-seq and only contrasted obtained transcriptome data from GFP-positive nuclei of all seven tissues (Supplemental Figure 3 and Supplemental Data Set 2). After we classified 4 out of 21 datasets as outliers based on principle component analysis (PCA) and correlation analysis (Supplemental Figure 3), we kept 17 datasets from which replicates clustered and correlated as expected (*n* = 2 or 3, Figure 3A and B; Supplemental Data Set 2). This decision was also based on the observation that the normalized read counts for some genes were not in line with their known gene expression profiles in the outlier samples (Supplemental Figure 3C and D) and that the reduced set of data better recapitulated the expression profiles of characterized genes. However, it is worth noting that identifying one out of three replicates as an outlier is not well-supported by statistical means. Therefore, we also provide the count tables as well as the raw data of the eliminated samples, allowing our processing to be reassessed (Supplemental Data Set 2). Confirming the biological relevance of the remaining profiles, the xylem-related (*PXY*_pro_, *NST3*_pro_, *VND7*_pro_) and phloem-related (*SMXL5*_pro_, *APL*_pro_) datasets showed high correlation coefficients among each other but belonged to two different major branches within the correlation plot (Figure 3B).

Overall, among the 37,051 Arabidopsis protein-coding and noncoding genes (Cheng et al., 2017), 25,679–33,949 genes were expressed in various tissues (Transcripts Per Million, TPM >1), indicating that our RNA-seq analyses had sufficient coverage (Table 1). The average percentage of reads mapping to introns compared with reads mapping to exons was 14.4–24.3%, which was higher than the 7.7% average found in our previous whole RNA-seq analyses of the second internode (Brackmann et al., 2018). This difference suggests that nucleus-derived RNA contains a higher proportion of nonprocessed transcripts compared with RNA derived from whole tissues (Supplemental Table 1). In contrast, the average percentages of reads mapping to intergenic regions versus exons were comparable (Supplemental Table 1), demonstrating that the observed difference in the percentage of intron-associated reads did not result from contaminations by genomic DNA.

**Table 1:** Summary of RNA-seq results

Datasets	Average number of detected gene activities (TPM > 1)	Percentage of all the annotated genes considered in this study (37,051 genes) (%)
FANS		
*NST3*_pro_	25,679	69.3
*VND7*_pro_	26,358	71.1
*PXY*_pro_	28,141	76.0
*SMXL5*_pro_	34,949	94.3
*APL*_pro_	33,233	89.7
*SCR*_pro_	27,716	74.8
*LTP1*_pro_	32,951	88.9
Whole organ		
Brackmann et al., 2018	18,033	48.7
LCM		
Phloem cap	17,393	46.9
Pith	15,127	40.8
Vascular bundle	16,845	45.5

Summary of RNA-seq results for the different internode tissues harvested by FANS, for the whole organ by conventional RNA extraction (Brackmann et al., 2018), and collected by LCM. *n* = 2 or 3 for each dataset.

**Figure 4:**
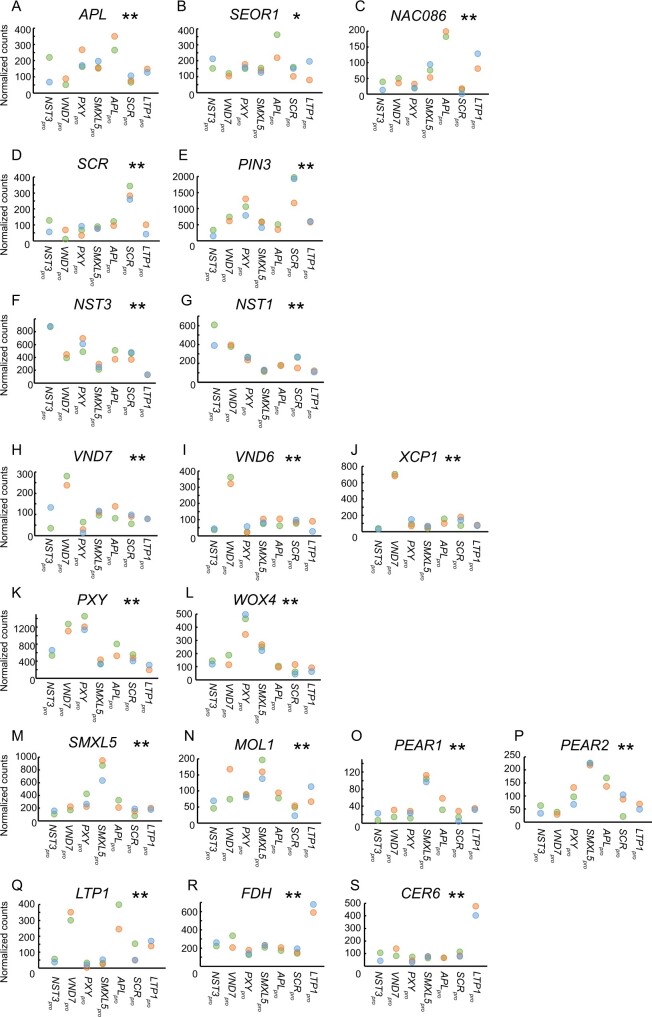
Comparison of transcriptome datasets from seven stem tissues derived from FANS. (A–S) Normalized gene read counts of the indicated genes among seven different tissues displayed for each replicate individually. *P < 0.05 and **P < 0.01, respectively, in the LRT. Please note that the null hypothesis to be rejected in the LRT is that genes have similar expression patterns among the seven different tissues.

When we compared gene expression levels in different tissues, reads from the *APL*, *SCR*, *NST3*, *VND7*, *PXY*, and *SMXL5* genes were, as expected, over-represented in samples derived from GFP-positive nuclei from the respective *GENE*_pro_*:H4-GFP* lines (Figure 4, A, D, F, H, K, and M). Moreover, *APL* expression peaked together with *SIEVE ELEMENT OCCLUSION-RELATED1* (*SEOR1*) and *NAC DOMAIN-CONTAINING PROTEIN86* (*NAC086*), which are known to be expressed in phloem cells (Froelich et al., 2011; Furuta et al., 2014; Figure 4B and C). Reads of the starch sheath-expressed gene *PIN-FORMED3* (*PIN3*) (Friml et al., 2002) were also most abundant in *SCR*_pro_-positive nuclei (Figure 4D and E). Likewise, reads from *NST1*, which is known to be expressed in fiber cells (Mitsuda et al., 2005), showed maximum abundance in *NST3*_pro_-positive nuclei (Figure 4F and G), and reads from *VND6* (the closest homologue of *VND7*) (Zhong et al., 2008) and its downstream target *XYLEM CYSTEINE PROTEASE1* (*XCP1*) (Zhong et al., 2010; Yamaguchi et al., 2011) showed the highest activity in *VND7_pro_*-positive nuclei (Figure 4H, I, and J). The activity of *WUSCHEL RELATED HOMEOBOX4* (*WOX4*), whose expression domain is congruent with the *PXY* expression domain (Hirakawa et al., 2008; Suer et al., 2011; Brackmann et al., 2018; Shi et al., 2019), also peaked in *PXY*_pro_-positive nuclei (Figure 4K and L). In addition, as expected, the activities of *MORE LATERAL GROWTH1* (*MOL1*), *PHLOEM EARLY DOF1* (*PEAR1*), and *PEAR2*, which are expressed in *SMXL5*_pro_-positive cells (Gursanscky et al., 2016; Miyashima et al., 2019), peaked in *SMXL5_pro_*-positive nuclei (Figure 4M–P). Although *LTP1* reads were not over-represented in *LTP1*_pro_-positive nuclei (Figure 4Q), the reads of *FIDDLEHEAD* (*FDH*) and *ECERIFERUM6* (*CER6*), which are known to be expressed in the epidermis (Yephremov et al., 1999; Pruitt et al., 2000; Hooker et al., 2002), were most abundant in *LTP1*_pro_-positive nuclei (Figure 4R and S). Finally, 32 out of 40 genes that previously showed high activity levels in the stem epidermis (Suh et al., 2005) had higher normalized read counts in the *LTP1*_pro_-derived dataset compared with the overall tissue average, and the reads of 23 of these 40 genes were indeed most abundant in *LTP1*_pro_-positive nuclei (Supplemental Figure 4), further verifying that we succeeded in determining the transcriptome of the epidermis.

### Transcriptome dataset of the phloem cap and pith

**Figure 5:**
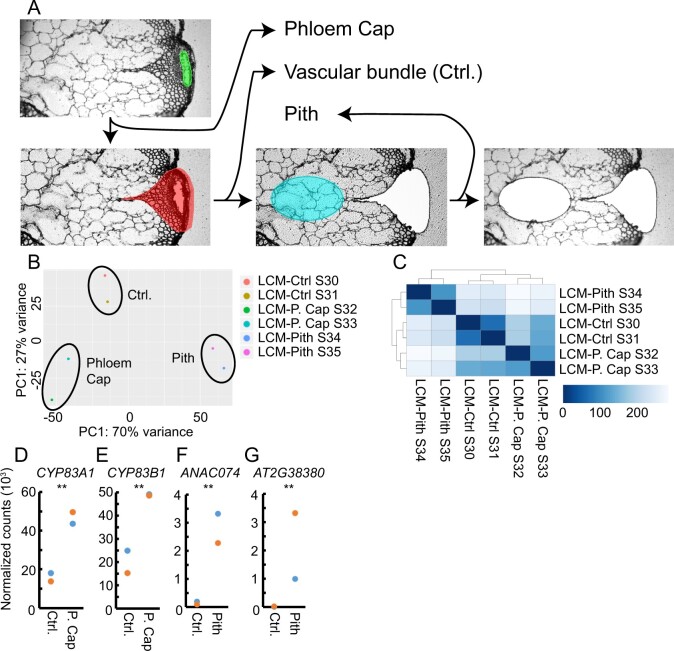
Transcriptome analysis of the phloem cap and the pith using laser capture microdissection (LCM). (A) Strategy of tissue collection using LCM shown for one individual sample. (B) PCA of log-transformed normalized read counts of each LCM-derived RNA-seq dataset. (C) Heatmap displaying the statistical distance between each RNA-seq dataset. Two replicates were obtained from each tissue type. Euclidean distance [arbitrary unit] between samples was calculated using the *dist* function in R, based on the log2-transformed normalized read counts. (D–G) Normalized gene read counts comparing phloem cap/pith and the vascular bundle (Ctrl.). **P < 0.01 in Wald tests.

Because no reliable tissue promoters were available for the pith and phloem cap, we employed LCM to determine transcriptome profiles of these tissues. To this end, we first collected the phloem cap and pith, i.e., the central region of the stem proximal to the vascular bundles (Figure 1B), followed by the remaining vascular bundle, which we harvested for comparison (Figure 5A, *n* = 2 replicates). We then extracted RNA from the tissues, amplified mRNA, and analyzed it by RNA-seq (Figure 5A;  Supplemental Data Set 3). Replicates generated from each sample type grouped together in PCA plots, confirming the reliability of sample preparation (Figure 5B). Moreover, correlation analyses showed that the phloem cap profile and the profile from the remaining vascular bundle were more similar to each other than to the pith profile (Figure 5C). This finding was expected considering the high spatial and ontogenetic relatedness between the vascular bundle and the phloem cap.

On average, we detected 15,127–17,393 expressed genes in the different LCM-derived sample types (TPM > 1), suggesting a lower coverage compared with our FANS-based analyses (Table 1). The average number of reads mapping to introns was 6.5% that of exons, a value in a similar range to the 7.7% found previously during standard RNA-seq analyses of the Arabidopsis stem (Brackmann et al., 2018; Supplemental Table 1). Supporting the notion that our profiling was reliable, reads from the *CYTOCHROME P450 83A1* (*CYP83A1*) and *CYP83B1* genes, which function in the indole and aliphatic glucosinolate biosynthetic pathways and are expressed in the phloem cap (Nintemann et al., 2018), were significantly over-represented in phloem cap samples (Figure 5D and E). In addition, reads from *ANAC074* and *AT2G38380*, which are known to be expressed in the pith (Fujimoto et al., 2018; Schürholz et al., 2018), were over-represented in pith-derived samples (Figure 5F and G).

### Identification of genes with tissue-associated expression patterns

**Figure 6:**
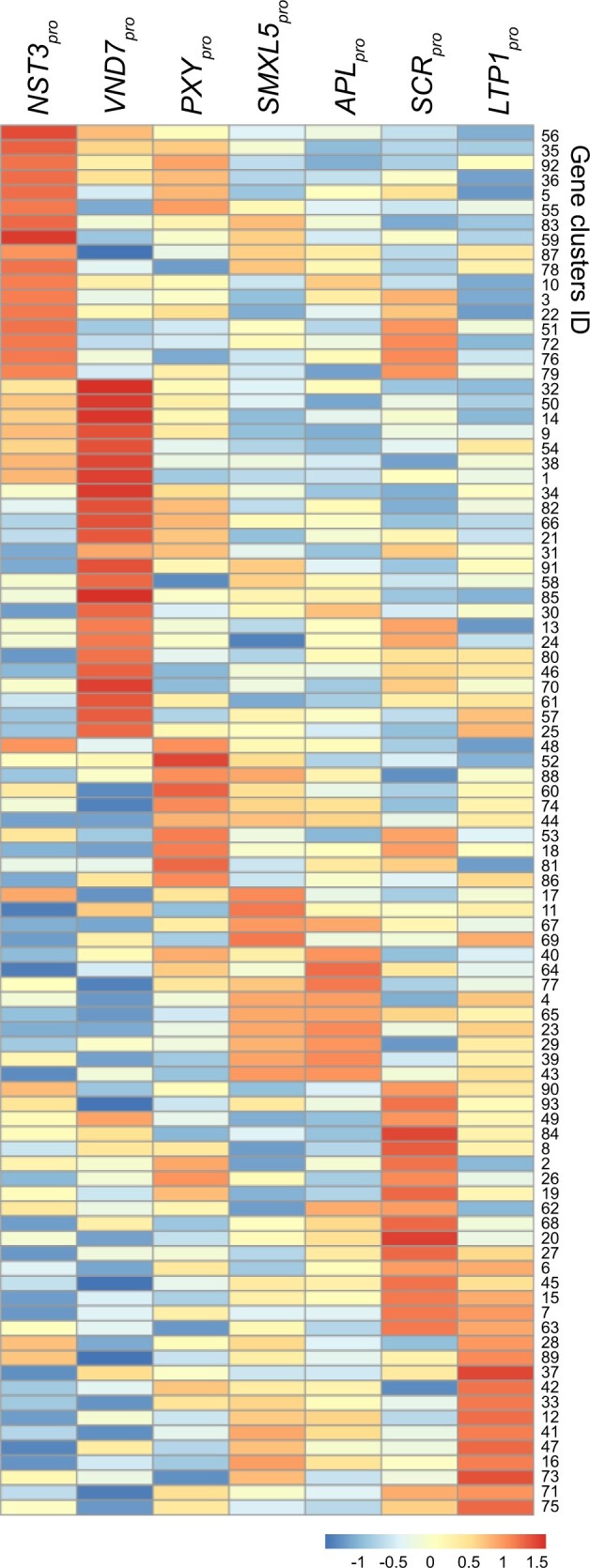
Clusters of differentially expressed genes among inflorescence stem tissues determined by FANS/RNA-seq. Heat map presenting relative gene activities among seven different tissues in each gene cluster. Relative gene expression values are colored according to the color scale at the bottom. Genes were clustered based on their expression pattern among seven tissues. See Supplemental Figure 5 for more detailed activity profiles of each cluster.

To identify genes with differential expression among the different tissue types, we applied the DESeq2 software package and the likelihood ratio test (LRT) to our FANS-derived datasets (Love et al., 2014). Based on this analysis, we classified 14,063 genes as significantly differentially expressed (SDE) genes among the seven tissues (Supplemental Data Sets 4 and 5; BH adjustment of *P*-value in LRT < 0.01). Based on their activity profiles, SDE genes were categorized into 93 clusters using hierarchical clustering (Figure 6;  Supplemental Figure 5 and Supplemental Data Set 6). The clusters contained 9–1,271 genes, with an average of 151 genes that, in most cases, were strongly active in one tissue and less active in all other tissues (Figure 6). The notable exceptions in this regard were developing (*SMXL5*_pro_) and differentiated (*APL*_pro_) phloem cells. In line with their strong ontogenetic relationship, genes very active in one of the two tissues were often very active in the second one (Figure 6). In contrast, clusters containing genes that were very active in fiber cells were mostly distinct from clusters containing genes whose activity was high in developing vessel elements. Similarly, most genes active in developing phloem cells (*SMXL5*_pro_) were distinct from genes active in developing xylem cells (*PXY*_pro_). These results suggest that, although these cell types partly originate from the same procambial precursors (Shi et al., 2019), they quickly establish very distinct profiles.

To identify genes that are mostly active in the phloem cap, we compared the phloem cap-derived dataset with the dataset derived from the remaining vascular bundle area. This comparison resulted in the identification of 575 phloem cap-associated genes (Supplemental Data Set 7, BH adjustment of *P*-value in Wald test < 0.01, fold change >2). As expected (Xu et al., 2019), among this group of genes, the gene ontology (GO) term glucosinolate biosynthetic process (GO:0019758) was over-represented (Supplemental Data Set 8). For the pith, we identified 1,633 genes whose reads were significantly over-represented in pith-derived samples compared with samples from the vascular bundle area (Supplemental Data Set 8, BH adjustment of *P*-value in Wald test <0.01, fold change >2). GO term analysis of this group of genes identified the term cell death (GO:0008219) as over-represented (Supplemental Data Set 8). This is in accordance with the finding that programed cell death is a characteristic of pith cells (Fujimoto et al., 2018). Taken together, these results demonstrate that our LCM-based transcriptomes recapitulated the expression profiles of characterized genes and provide informative insights into phloem cap and pith-specific cellular processes.

### Tissue-specific mRNA profiling identifies cell type-associated promoters

**Figure 7:**
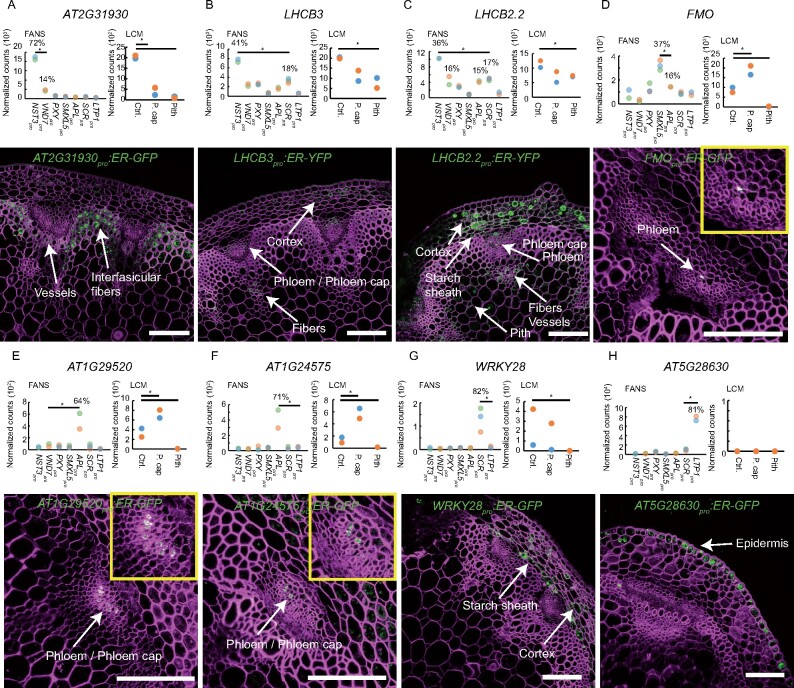
Validation of the estimated gene expression patterns found in FANS-derived datasets. (A–H) Upper panels: normalized read counts among the different FANS- and LCM-derived datasets are shown for each gene indicated (*AT2G31930*, *LIGHT-HARVESTING CHLOROPHYLL BINDING PROTEIN 3* (*LHCB3*), *LHCB2.2*, *FLAVIN-CONTAINING MONOOXYGENASE* (*FMO*), *AT1G29520*, *AT1G24575*, *WRKY28*, *AT5G28630*). Percentages given in the FANS chart (left) indicate the ratio between the average number of the normalized read counts in the selected nucleus type and the average number of the normalized read counts found in all seven nucleus types. Asterisks indicate significant differences between the highest value and the second highest value in the FANS charts (*P* < 0.01 in Wald test), and significant differences compared with the control tissue in the LCM charts (*P* < 0.01 in Wald test). Bottom panels: confocal microscopy images from each indicated promoter-reporter line. The endoplasmic reticulum (ER)-targeted fluorescent signal is shown in green. The cell wall was stained with Direct Red 23 and is visualized in magenta. Scale bars: 100 *µ*m. A single focal plane is shown, except in H, where a maximum intensity projection is shown. At least two independent transgenic plant lines for each promoter-reporter were investigated. Detected signals are indicated by white labels. Autofluorescence in the green channel was sometimes detected in the cortex (e.g. in D and F).

To characterize the power of our SDE estimations, we first turned to the FANS/RNA-seq datasets. Here we selected 23 promoters from genes that showed at least two-times more normalized reads in one tissue compared with any other tissue and exhibited a significant difference between the highest tissue-specific value and the second highest value (Supplemental Data Set 9, fold change >2, Wald test, *P* < 0.01). Of the 23 promoter-reporters tested, eight behaved as predicted, with predominant activities in fibers, distal cambium, phloem, starch sheath, and epidermis, respectively (Figure 7). Because the cortex was not included in this study, we did not consider cortex-associated gene expression (as for example observed for the *LHCB2.2_pro_:ER-YFP* reporter, Figure 7C) as being contradictive. Seven promoter-reporters showed predominant activities in tissues different from predicted ones (Supplemental Figure 6), and the activities of eight promoter-reporters were barely detectable in any tissue (Supplemental Figure 7).

**Figure 8:**
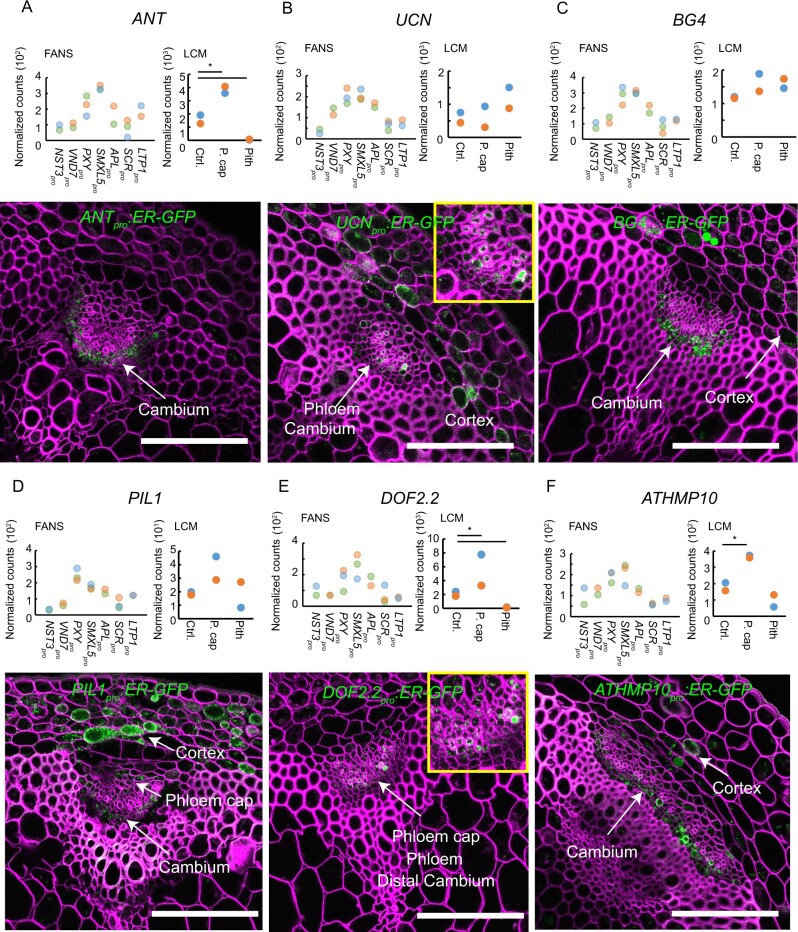
Characterization of promoter activities of genes estimated to be specifically expressed in the cambium region (cluster 88). (A–F) Upper panels: normalized read counts among FANS- and LCM-derived datasets are shown for each gene indicated (*ANT*, *UCN*, *BG4*, *PIL1*, *DOF2.2*, *ATHMP10*). Asterisks indicate significant differences between the highest value and the second highest value in the FANS-derived charts (*P* < 0.01 in Wald test, not detected), and significant differences compared with the control tissue in LCM-derived charts (*P* < 0.01 in Wald test). Bottom panels: confocal microscopy images for each indicated promoter-reporter line. The endoplasmic reticulum (ER)-targeted fluorescent signal is shown in green. The cell wall was stained with Direct Red 23 and is visualized in magenta. Scale bars: 100 *µ*m. A single focal plane is shown in all images. At least two independent transgenic plant lines for each promoter-reporter were investigated. Detected signals are indicated by white labels.

Next, we explored the predictive power of SDE clusters associated with more than one tissue. Conveniently, we recently showed that cambium stem cells are specifically localized in a narrow domain with overlapping *PXY* and *SMXL5* expression (Shi et al., 2019). Therefore, we focused on cluster 88, where gene expression was detected in both the *PXY*_pro_ and *SMXL5*_pro_ domains (Figure 6), expecting to find that genes specific for cambium stem cells were part of this cluster. Indeed, *AINTEGUMENTA* (*ANT*), which is expressed in cambium stem cells in roots (Randall et al., 2015; Smetana et al., 2019; Zhang et al., 2019), was part of cluster 88, and analysis of *ANT_pro_:ER-GFP-HDEL* promoter-reporter expression revealed that *ANT* was also expressed in the cambium of inflorescence stems (Figure 8A). From the 37 genes in cluster 88, we selected five genes, including *UNICORN* (*UNI*), *BIG GRAIN 4* (*BG4*), *PHYTOCHROME INTERACTING FACTOR 3-LIKE 1* (*PIL1*), *DOF 2.2*, and *HEAVY METAL-ASSOCIATED PROTEIN 10* (*ATHMP10*), and generated promoter-reporter lines to analyze their activity patterns. In all five reporter lines, we detected signals in the cambium domain, including cambium stem cells, as indicated by our FANS-derived datasets (Figure 8B–F), pointing to the high predictive power of our cluster analysis for estimating gene expression patterns.

**Figure 9:**
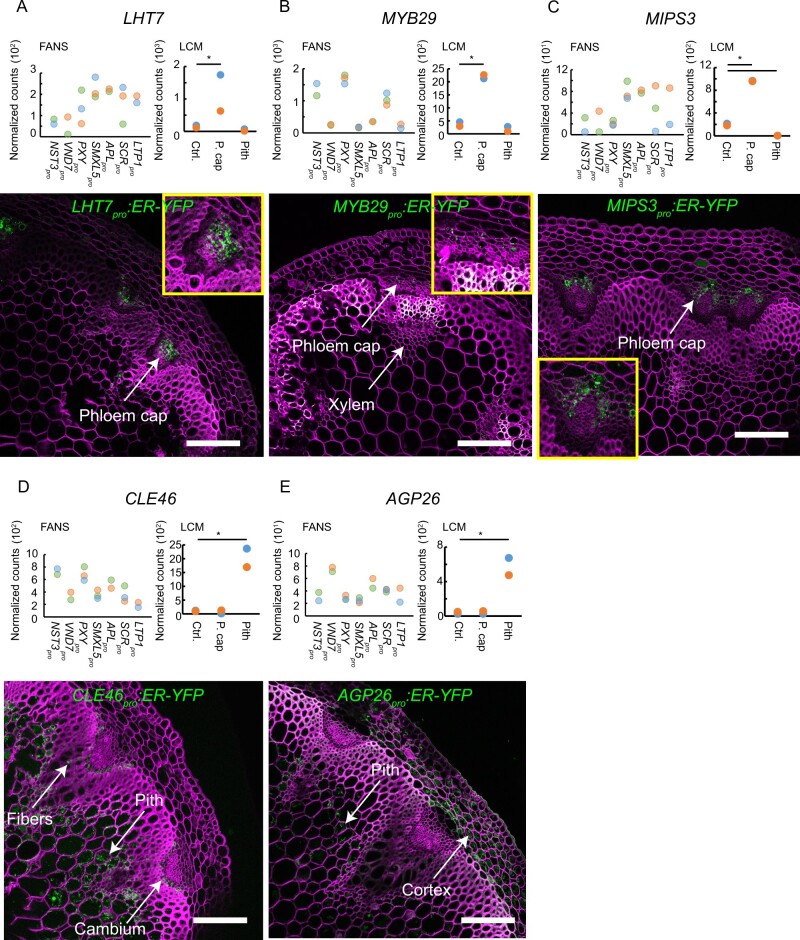
Validation of estimated gene expression patterns found in LCM-derived datasets. (A–E) Upper panels: normalized read counts among different FANS- and LCM-derived datasets are shown for each gene indicated (*LHT7*, *MYB29*, *MIPS3*, *CLE46*, *AGP26*). Asterisks indicate significant differences between the highest value and the second highest value in the FANS-derived charts (*P* <0.01 in Wald test, not detected), and significant differences compared with the control tissue in LCM-derived charts (*P* < 0.01 in Wald test). Bottom panels: confocal microscopy images for each indicated promoter-reporter line. The endoplasmic reticulum (ER)-targeted fluorescent signal is shown in green. The cell wall was stained with Direct Red 23 and is visualized in magenta. Scale bars: 100 *µ*m. A single focal plane is shown in all images. At least two independent transgenic plant lines for each promoter-reporter were analyzed. Detected signals are indicated by white labels.

For the LCM-derived datasets, we selected five genes with significantly higher expression values in the phloem cap or the pith compared with the vascular bundle (Supplemental Data Set 7, BH adjustment of *P *<* *0.01 in the Wald test, fold change >2). When analyzing respective promoter-reporter lines, we found that *LYS/HIS TRANSPORTER 7* (*LHT7*), *MYB29*, and *MYO-INOSITOL-1-PHOSPHATE SYNTHASE ISOFORM 3* (*MIPS3*) reporters were active in the phloem cap region (Figure 9A–C), and *CLAVATA3/ESR-RELATED PROTEIN 46* (*CLE46*) and *ARABINOGALACTAN PROTEIN 26* (*AGP26*) reporters were active in the pith (Figure 9D–E). In summary, we confirmed the association of reporter activities with the predicted tissues in 56% (19/34) of the cases selected from the different datasets. Based on these results, and considering that we only included a certain region upstream of the respective start codons into our reporters, we conclude that our tissue-specific transcriptional profiles provide a reliable estimate of gene expression patterns in inflorescence stem tissues.

### Pairwise comparison of wood-related cell types reveals distinct physiological signatures

**Figure 10:**
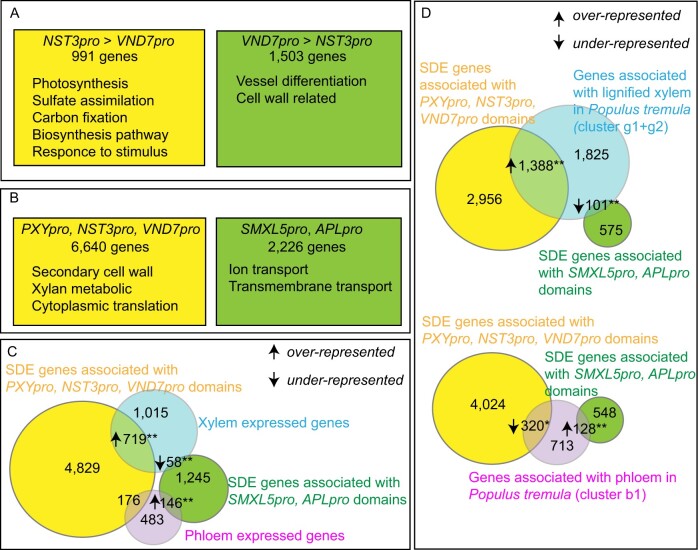
Pairwise comparison of *NST3*_pro_ and *VND7*_pro_ domains, and comparisons of FANS-derived datasets with previously published xylem or phloem-related genes from Arabidopsis roots and aspen stems. (A) Summary of pairwise comparison of *NST3*_pro_ and *VND7*_pro_ domains in the FANS-derived datasets. The numbers of SDE genes (14,063 genes in total) highly expressed in the *NST3*_pro_ or the *VND7*_pro_ domain are shown (BH adjustment of *P*-value in Wald test < 0.01, fold change > 2). Over-represented GO terms for each gene list are shown (*P* < 0.05 in Fisher's Exact with Bonferroni correction). For detailed GO terms enrichment analysis results, please consult Supplemental Data Set 11. (B) Comparison of *NST3*_pro_, *VND7*_pro_, *PXY*_pro_-associated and *SMXL5*_pro_, *APL*_pro_-associated genes as determined from the FANS-derived datasets. SDE genes (14,063 genes in total) associated with the specified domains were selected from the clustering analysis, and over-represented GO terms for individual comparisons are presented (*P* < 0.05 in Fisher's exact test with Bonferroni correction). Detailed GO term enrichment analysis results are shown in Supplemental Data Set 13. (C) Comparison of *NST3*_pro_, *VND7*_pro_, *PXY*_pro_-associated and *SMXL5*_pro_, *APL*_pro_-associated genes with genes associated with the xylem or the phloem of roots derived from Brady et al., 2007 (23,615 genes in total). (D) Comparison of *NST3*_pro_, *VND7*_pro_, *PXY*_pro_-associated and *SMXL5*_pro_, *APL*_pro_-associated genes with genes associated with the lignified xylem or the phloem in aspen stems derived from AspWood (in total 13,530 *Populus tremula* genes annotated to Arabidopsis genes after de-duplication). The gene lists were obtained from annotated Arabidopsis genes and de-duplication. ^*^P < 0.01 and ^**^P < 1e−05 in Fisher’s exact test, respectively. Full gene lists used for the comparisons are shown in Supplemental Data Set 12.

To investigate whether pairwise comparisons of the analyzed tissues were also useful for predicting tissue-specific processes, we directly contrasted gene expression profiles from functionally and ontogenetically related tissues. Fibers (*NST3*_pro_) and xylem vessels (*VND7*_pro_) both determine wood properties in angiosperms and show distinct morphologies which are important for conducting their specific functions (Evert, 2006). Accordingly, their functional specialties are reflected in very distinct expression profiles (Figure 6). Comparing *NST3*_pro_- and *VND7*_pro_-derived datasets, we identified 991 and 1,503 genes as being predominantly expressed in *NST3*_pro_-positive and *VND7*_pro_-positive nuclei, respectively (Figure 10A;  Supplemental Data Set 10, BH adjustment of *P*-value in Wald test <0.01, fold change >2). Using GO enrichment analysis (Mi et al., 2019), we also found that the terms photosynthesis (GO:0015979), sulfate assimilation (GO:0000103), and response to cytokinin (GO:0009735) and other stimulus-related genes are significantly over-represented within the group of *NST3*_pro_-derived genes, whereas the terms xylem vessel differentiation (GO:0048759) and cell wall biogenesis (GO:0042546) were, as expected, over-represented within classifications of *VND7*_pro_-associated genes (Figure 10A;  Supplemental Data Set 11).

### Comparisons with previously published datasets allows organ- and species-related gene activities to be determined

To determine to what extent gene expression patterns in vascular tissues are shared between roots and stems, we compared the xylem-related *NST3*_pro_, *VND7*_pro_, and *PXY*_pro_-associated SDE gene clusters and the phloem-related *SMXL5*_pro_ and *APL*_pro_-associated clusters with tissue-specific gene expression datasets from roots (Brady et al., 2007). The combined group of *NST3*_pro_, *VND7*_pro_, and *PXY*_pro_-associated genes consisted of 6,640 genes from 49 clusters. GO term enrichment analyses identified the terms secondary cell wall biogenesis (GO:0009834) and xylan metabolic process (GO:0045491) as over-represented among these genes [Figure 10B;  Supplemental Data Sets 12 and 13; Fisher’s exact test *P *<* *0.05 {Bonferroni-corrected}]. The combined group of *SMXL5*_pro_ and, *APL*_pro_-associated genes consisted of 2,226 genes from 13 clusters, among which the terms ion transport (GO:0006811) and transmembrane transport (GO:0055085) were over-represented in GO term enrichment analyses [Figure 10B;  Supplemental Data Sets 12 and 13; Fisher’s exact test *P *<* *0.05 {Bonferroni-corrected}]. When we compared these groups with the genes expressed in the xylem or phloem of roots (combined S4, S18, and JO121 datasets for xylem and combined SUC2, S32, APL, and S17 datasets for phloem (Lee et al., 2006; Brady et al., 2007)), we found that xylem-associated genes from roots were significantly over-represented among *NST3*_pro_, *VND7*_pro_, and *PXY*_pro_-associated genes from stems (*P *<* *1e-05 in Fisher’s Exact test; Figure 10C;  Supplemental Data Set 12). Likewise, phloem-associated genes from roots were significantly over-represented among *SMXL5*_pro_ and, *APL*_pro_-associated genes (*P *<* *1e-05 in Fisher’s Exact test; Figure 10C;  Supplemental Data Set 12). This observation suggests that a substantial number of genes are shared between vascular tissues of primary roots and stems, but there are also large differences in the molecular signatures when comparing vascular tissues from both organs.

Next, we compared our data with tree-derived data available at AspWood, a platform that provides tissue-specific transcriptome profiles from aspen (*Populus tremula*) with a high spatial resolution along the radial sequence of tissues of the cambium domain (Sundell et al., 2017). First, we extracted phloem-specific (b1 cluster) and lignified xylem-specific (g1, g2 clusters) genes and, after removing duplicates, obtained the corresponding Arabidopsis gene annotations from AspWood (Sundell et al., 2017) (b1: 1,161 genes, g1, g2: 3,314 genes, Supplemental Data Set 12). Comparing these genes with our clusters, we found that our *NST3*_pro_, *VND7*_pro_, and *PXY*_pro_-associated genes significantly overlapped with lignified xylem-associated genes, and *SMXL5*_pro_ and, *APL*_pro_-associated genes significantly overlapped with phloem-associated genes from aspen (*P *<* *1e-05 in Fisher’s Exact Test; Figure 10D;  Supplemental Data Set 12). As expected, these data demonstrate that gene expression profiles from vascular tissues are largely conserved among species.

### Promoter analyses identify tissue-related transcription factor binding sites

Networks of transcription factors are vital for establishing tissue-specific gene expression profiles, thereby determining cellular behavior (Gaudinier and Brady, 2016). Taking advantage of our newly identified SDE genes, we sought to identify transcription factor binding regions over-represented in the promoters of genes with similar expression patterns. To this end, we first assessed the significance of the overlap between the promoters of genes from each SDE cluster and the binding profiles of 387 transcription factors derived from massive DNA affinity purification sequencing (DAP-Seq; O'Malley et al., 2016). Among the 31 clusters, which each contained more than 150 genes, we identified significant over-representation of transcription factor binding profiles in 13 clusters [Table 2;  Supplemental Data Set 14; *P *<* *8.8e-05 {Bonferroni adjusted threshold of 0.05} in Fisher’s exact test]. Enrichment for the overlap with transcription factor binding profiles within the upstream regions of phloem cap-associated genes identified six putative transcriptional regulators from the NAM, ATAF1/2 AND CUC2 (NAC), MYELOBLASTOSIS (MYB), and REPRODUCTIVE MERISTEM (REM) families (Table 2; Supplemental Data Set 14). Of these families, the poorly studied ANAC028 transcription factor was mostly active in the phloem cap itself (Supplemental Data Set 7), in addition to being expressed in the protophloem of roots (Brady et al., 2007). Transcription factor binding region enrichment analyses for pith-associated genes predicted a set of 61 potential regulators (Table 2; Supplemental Data Set 14). Of these, homeodomain transcription factors (ATHBs), PHAVOLUTA (PHV), and the APETALA 2/ETHYLENE RESPONSE FACTOR (AP2/ERF) members ERF34 and ERF38 were among the transcription factors specifically expressed in the pith (Supplemental Data Set 7).

**Table 2:** Over-represented transcription factor (TF) binding regions in promoters from SDE cluster genes derived from FANS and in promoters from phloem cap- or pith-associated genes derived from LCM

Gene list	Number of genes	Region with high expression	Number of over-represented TF binding regions	Associated TFs^a^
FANS cluster ID				
8	436	*SCR*_pro_	52	CBF1-4; BIM2; CAMTA1, 5; GBF3; ABI5; RTV1; WRKY
7	245	*SCR*_pro_, *LTP1*_pro_	41	CAMTA1, 5; WRKY
19	181	*SCR*_pro_, *PXY*_pro_	35	CAMTA1, 5; WRKY
24	358	*VND7*_pro_, *SCR*_pro_	20	TINY; AREB3; GBF3; bZIPs; ERF38; CAMTA5
14	528	*VND7*_pro_	17	VND1-2; SMB; MYB; GT2
15	223	*SCR*_pro_, *LTP1*_pro_	16	CBF1-4; WRKY
21	220	*VND7*_pro_, *PXY*_pro_	15	CAMTA1; PHV; LMI1; ATHB
22	477	*NST3*_pro_, *SCR*_pro_	4	ABF2; HY5; MYB55; MYB83
5	445	*NST3*_pro_, *PXY*_pro_	3	TBP3
3	192	*NST3*_pro_, *SCR*_pro_	1	ABI5
38	265	*NST3*_pro_, *VND7*_pro_	1	AT1G49560
41	790	*LTP1*_pro_, *SMXL5*_pro_	1	ATHB13
49	183	*SCR*_pro_, *VND7*_pro_	1	ERF38
LCM				
Phloem cap	576	Phloem cap	6	NAC, MYB
Pith	1634	Pith	61	ATHBs; PHV; ERF34,38; Indeterminate-domain (IDD) proteins

^a^ Only a fraction of the whole list is shown. Please consult Supplemental Data Set 14 for the enrichment fold value and the full list.

The set of newly identified transcription factors or their larger families contains promising candidates for determining tissue-specific signatures. In cluster 14, for example, which was associated with developing vessel elements, we found potential binding regions for 17 different transcription factors that were over-represented in the respective promoters. These transcription factors included VASCULAR-RELATED NAC-DOMAIN1 (VND1, 4.3-fold enrichment) and VND2 (4.6-fold enrichment; Table 2;  Supplemental Data Set 14). Because these transcription factors are expressed in developing vessels where they promote secondary cell wall formation (Zhou et al., 2014), our analysis indeed holds the potential to identify tissue-specific regulators in the inflorescence stem. In turn, in a large number of clusters, no over-representation of binding sites was detected, suggesting that, in general, established tissues do not depend on a small set of transcriptional regulators to maintain their identity.

## Discussion

Organs are functional units composed of different tissues that determine distinct aspects of their performance. Here, by combining FANS and LCM-based mRNA harvesting with RNA-seq analyses, we established a tissue-specific gene expression atlas of the primary Arabidopsis inflorescence stem. In addition to being accessible via the Gene Expression Omnibus (GEO) depository (Barrett et al., 2013) under accession number GSE142034, data for genes of interest can be accessed via a website, allowing expression profiles to be easily extracted (https://arabidopsis-stem.cos.uni-heidelberg.de/). We also provide lists of tissue-specific genes derived from our FANS and LCM transcriptome data whose expression was estimated to be specific for a single tissue (Supplemental Data Set 15). For some tissues analyzed by FANS/RNA-seq (*PXY*_pro_, *SMXL5*_pro_, *APL*_pro_), these lists are rather short due to a high degree of shared gene expression patterns with other tissues. To extract genes with more complex expression patterns, the provided gene clusters (Figure 6;  Supplemental Figure 5 and Supplemental Data Set 6) would be more suitable.

Several observations underline the robustness of our mRNA profiling. First, the activities of six out of seven genes whose promoters were used for tissue-specific nucleus labeling were found to peak in the respective tissues. This observation suggests that the chosen promoters mostly recapitulate the expression patterns of endogenous genes. Moreover, it shows that nuclei maintain a sufficient amount of their mRNA content during the isolation process and that the contamination by cytosolic mRNA, which is released during tissue disruption, is low. The finding that *LTP1* activity did not peak in *LTP1*_pro_-positive nuclei may be an example of discrepancies between the activity pattern of our chosen *LTP1* promoter and the distribution of endogenous *LTP1* mRNA. In this context, it is important to be aware that the stability of *LTP1* mRNA or the possible migration of this mRNA from cell to cell might be very different compared with *H4-GFP* mRNA driven by the *LTP1* promoter. This difference may result in discrepancies between the patterns of H4-GFP protein accumulation and *LTP1* mRNA accumulation. However, our comparison with previous datasets strongly suggested that we succeeded in isolating epidermis-specific mRNA.

Interestingly, we identified many more genes as being expressed in specific tissues by directly comparing the different GFP-positive nucleus populations than by comparing two respective sets of GFP-positive and GFP-negative nuclei (Supplemental Data Set 1). We speculate that this finding is due to the higher heterogeneity of GFP-negative nuclei compared with GFP-positive nuclei and the higher number of GFP-positive samples in the former comparison. Therefore, we propose that comparing GFP-positive nucleus populations is superior to comparing GFP-positive and GFP-negative samples when determining gene expression profiles.

The second observation that supports robustness of the newly obtained profiles is that the promoters of genes we predicted to have a distinct spatial pattern recapitulated, in large, these patterns. In addition to the possible reasons resulting in differences between the accumulation of endogenous transcripts and the H4-GFP protein discussed above, it is important to note that the promoter regions upstream of the respective start codons, which we chose to drive the fluorescent reporters, may miss regulatory elements that substantially influence the expression of the endogenous genes (Raatz et al., 2011). Therefore, a certain level of differences between FANS/LCM-derived profiles and reporter activities are expected and do not necessarily argue for a low predictive power of our transcriptional profiles. As a third observation arguing for the relevance of our profiles, the activity patterns of genes and pathways known to be associated with certain tissues were reflected in our datasets. Taken together, we conclude that the obtained expression data provide a realistic picture of gene activities in the Arabidopsis inflorescence stem.

Although cytosolic and nuclear mRNA populations may differ due to the tight regulation of nuclear export or differences in mRNA homeostasis (Yang et al., 2017; Choudury et al., 2019), our results indicate that the gene expression profiles of mature plant organs can be faithfully characterized by profiling nuclear mRNA. Interestingly, our RNA-seq results show that the sensitivity of FANS/RNA-seq-based analyses is comparable or even higher than that of conventional profiling of whole organs by RNA-seq (Table 1). Compared with the profiling of mRNA from whole cells, there are several other advantages of fluorescence-based profiling of nuclear mRNA (Lake et al., 2017; Abdelmoez et al., 2018; Bakken et al., 2018; Wu et al., 2019). First, profiling nuclear mRNA allows differentiated plant tissues with prominent cell walls or heterogeneous protoplast sizes that can be labeled by genetically encoded fluorescent reporters to be analyzed. Thus, this method allows a broad range of tissues to be targeted regardless of enzymatic accessibility and without the use of morphological markers, which are required for tissue identification when using LCM. Second, following our procedure, tissue disruption and subsequent washing steps during nucleus extraction takes 30–40 min. Depending on cell wall properties, protoplast generation often requires more time (Birnbaum et al., 2003), which increases the risk of treatment-induced changes in transcript abundance. Third, nuclear mRNAs carry a higher ratio of unprocessed mRNA molecules compared with mRNA from whole cells (Lake et al., 2017; Abdelmoez et al., 2018; Wu et al., 2019). Due to a predictable rate of mRNA processing in nuclei, this feature can be used to calculate the actual transcription rates of individual genes (Gaidatzis et al., 2015; Lake et al., 2016). Therefore, nuclear RNA-seq datasets provide a different quality of information compared with RNA-seq datasets from whole cells. Fourth, compared with precipitation-based methods for nucleus isolation such as INTACT (Deal and Henikoff, 2010), thresholds for fluorescence-based nucleus sorting can be adequately set based on the fluorescence intensity. This feature not only provides flexibility in selecting distinct nuclei populations based on the level of reporter activity, but it might also allow a multitude of different fluorescent markers to be combined in order to collect distinct, highly specific nucleus populations from single plant lines.

Underlining the differences between nucleus-based profiling and profiling of RNA from whole cells, the LCM-derived datasets showed substantial differences from FANS-derived datasets even after normalization (Supplemental Figure 8). Due to these differences, the majority of Arabidopsis genes (75%) were classified as SDE genes when contrasting FANS-derived and LCM-derived datasets. Thus, we could not perform meaningful clustering to integrate all tissue-specific datasets across the different sample types. In addition to the different cellular compartments from which the RNA was isolated, the different sampling and processing methods most likely contribute to this lack of comparability, emphasizing the importance of similar experimental procedures in these types of experiments.

In summary, considering the importance of plant stems and their tissues for land plant evolution on the one hand (Xu et al., 2014) and biomass accumulation on the other hand (Yamaguchi and Demura, 2010), information about their specific gene expression profiles is certainly vital. Our datasets will allow researchers to formulate testable hypotheses about the activities of distinct pathways in individual stem tissues and their roles in determining overall plant performance.

## Materials and methods

### Plant material

*Arabidopsis thaliana* Col-0 plants were used in this study. The plants were grown in soil (Profi Substrat Classic CLT SM fein, Einheitserde, Sinntal-Altengronau, Germany, #10-00306-40) alone or mixed with perlite to 20% (PERLIGRAN Extra, Knauf Performance Materials, Dortmund, Germany) under short-day conditions (10-h light and 14-h darkness) for 3–5 weeks and transferred to long-day conditions (16-h light and 8-h darkness) for 3 weeks to induce reproductive growth. Light was provided by red and blue LED lights (GreenPower LED production DR/B LO, Phlips (Signify), Eindhoven, The Netherlands, #9290 004 871) at 70–120 *µ*M photosynthetically active radiation. The *SMXL5*_pro_*:H4-GFP* (*pIL53*) line and the *PXY*_pro_*:H4-GFP* (*pPS24*) lines were described previously (Shi et al., 2019). Other transgenic lines were generated by the floral dip method using *Agrobacterium tumefaciens* (Clough and Bent, 1998). Analyses were performed using homozygous lines, except for promoter-reporter line analyses, where plants from the T1 or T2 generation were used.

### DNA vector construction

An *H4-GFP*-containing construct was a gift from Daniel Schubert (Freie Universität Berlin, Germany). A vector containing the *ANT* promoter region (MT63, Schoof et al., 2000) was used as a template for PCR-based cloning (Supplemental Table 2). *NST3*_pro_*:H4-GFP* (*pMS59*), *VND7*_pro_*:H4-GFP* (*pTOM61*), *APL*_pro_*:H4-GFP* (*pPS02*), *SCR*_pro_*:H4-GFP* (*pPS20*), and *LTP1*_pro_*:H4-GFP* (*pPS16*) were cloned using the pGreen0229 vector (Hellens et al., 2000) as a backbone. Cloning of the *APL* promoter and terminator regions was described previously (Sehr et al., 2010). Primer sequences used to clone promoter and terminator regions of other genes and to amplify the *H4-GFP* sequence with the appropriate restriction enzyme sites are listed in Supplemental Table 2. Constructs for the YFP promoter-reporter lines were generated using a modified pGreen0229 vector as a backbone containing a sequence encoding the enhanced yellow fluorescent protein (EYFP) targeted to the endoplasmic reticulum (*ER-EYFP-HDEL*) (Haseloff et al., 1997) and the *AT4G24550* (*ADAPTOR PROTEIN-4 MU-ADAPTIN*; *AP4M*) terminator region generated by In-Fusion cloning (TaKaRa, Kusatsu, Japan) using previously described vectors as templates (Suer et al., 2011; Brackmann et al., 2018). The promoter region sequences were amplified using primers listed in Supplemental Table 2 and cloned into the backbone by In-Fusion cloning. Constructs for the GFP promoter-reporters were generated using the GreenGate system (Lampropoulos et al., 2013). Promoter region sequences were amplified using primers listed in Supplemental Table 2, cloned into the *pGGA000* vector, or directly synthesized as listed in Supplemental Data Set 16 by GENEWIZ (Leipzig, Germany). The vectors were then used for GreenGate reactions with the ER signal peptide sequence (*pGGB006*), the *GFP* sequence (*pGGC014*), the *HDEL* sequence (*pGGD008*), the *RBCS* terminator sequence (*pGGE001*), the BASTA resistance sequence (*pGGF001*), and the destination vector (*pGGZ003*) (Lampropoulos et al., 2013).

### Confocal microscopy

Free-hand cross-sections of the second bottom-most internodes of Arabidopsis inflorescence stems were generated using razor blades (Wilkinson Sword, High Wycombe, UK) and stained with 0.1% solution of Direct Red 23 (Hoch et al., 2005; Anderson et al., 2010; Ursache et al., 2018; 30% content powder, Sigma-Aldrich, St Louis, #212490) in Ca^2+^, Mg^2+^-free PBS. The sections were briefly washed in tap water and placed into a glass-bottom dish (ibidi, Gräfelfing, Germany, µ-Dish 35 mm, high, #81151). Images were captured using a Nikon A1 confocal microscope with a 25x water immersion objective lens (Nikon, Tokyo, Japan, Apo 25xW MP, 77220) and gallium arsenide phosphide detectors. GFP or YFP was excited by a 488-nm laser light, and Direct Red 23 was excited by a 561-nm laser light.

### Nucleus isolation

The second internodes of Arabidopsis inflorescence stems were collected in a Petri dish on ice. Each 2 g stem tissue sample was combined with 2-mL cold isolation buffer (20-mM Tris [pH = 7.5], 40-mM NaCl, 90 mM KCl, 2-mM ethylenediaminetetraacetic acid [pH = 8.0], 0.5-mM ethylene glycol-bis(β-aminoethyl ether)-N,N,N′,N′-tetraacetic acid (EGTA), 0.05% Triton X-100, 0.5-mM Spermidine, 0.2-mM Spermine, 15-mM 2-mercaptoethanol, 0.5-mM phenylmethylsulfonylfluorid, cOmplete Protease Inhibitor Cocktail [Roche, Basel, Switzerland, #11697498001]) supplemented with 10 *µ*L RiboLock RNase inhibitor (40 U/*µ*L) (ThermoFisher, Waltham, MA, #EO0381). The samples were chopped manually on ice for 10 min and transferred to a low binding tube (Protein LoBind Tube, Eppendorf, Hamburg, Germany, #0030 108.132) through a filter (CellTrics 50 *µ*m, Sysmex, Kobe, Japan, #04-004-2327). Nuclei were collected by centrifugation (1,000*g*, 10 min, 4°C) and gently washed twice with cold resuspension buffer (isolation buffer without Triton X-100). About 300 *µ*L of resuspension buffer supplemented with a final concentration of 10 *µ*g/mL Hoechst 33342 and 5-*µ*L RiboLock RNase inhibitor were added to the nuclei, which then were transferred to a tube through a filter (FALCON Round-Bottom Tube with Cell-Strainer Cap, Corning, NY, #352235) for sorting.

### Nucleus sorting

Nucleus sorting was performed on a BD FACSAria^TM^ IIIu cell sorter (Becton Dickinson, Franklin Lakes, NJ) using a 70-*µ*m sort nozzle. A sheath pressure of 70 psi and a drop drive frequency of 87 kHz were applied. GFP fluorescence was excited at 488 nm using a 20 mW blue laser and Hoechst fluorescence at 405 nm using a 50 mW violet laser. A 530/30 bandpass filter was used for GFP and a 450/40 bandpass filter for Hoechst detection. Autofluorescence at 405-nm excitation was detected using a 585/42 bandpass filter. Forward scatter (FSC) detector voltage was set to 55, and a 1.0 neutral density filter was used. Side scatter (SSC) detector voltage was set to 275, Hoechst detector voltage to 352, and GFP detector voltage to 379. Events were triggered on FSC using a threshold of 5,000 and Hoechst with a threshold of 400. All the events were first filtered by FSC and SSC (Supplemental Figure 2E, gate 1). Nuclei were then identified based on Hoechst-derived fluorescence. Doublets and aggregates were characterized by their higher Hoechst signal width values and were excluded from sorting by plotting the Hoechst signal width against the Hoechst signal area (Supplemental Figure 2F, gate 2). To exclude false-positive events due to autofluorescence in the yellow-green spectral range, values from the GFP detection channel were plotted against values from a yellow fluorescence detection channel, and only events with low yellow signal intensity were selected for sorting (Supplemental Figure 2G, gate 3). Next, the gate for selecting GFP-positive nuclei was set using wild-type plants as a reference (Supplemental Figure 2, H, J–O, gate 4). DNA content distribution was used to monitor the sorting procedure (Supplemental Figure 2I).

### RNA extraction, cDNA library preparation, and next-generation sequencing

About 15,000 nuclei per population were sorted into 750-*µ*l TRIzol reagent (ThermoFisher, Waltham, MA, #15596018), except for the *LTP1*_pro_-S21 sample, for which 10,800 nuclei were collected. RNA was precipitated and washed according to the manufacturer’s protocol and resuspended in 15-*µ*L water. One mircoliter of total RNA was used for mRNA-seq library construction using the Smart-seq2 protocol (Picelli et al., 2013; Hofmann et al., 2019). The resulting cDNA was quantified using a Qubit Fluorometer (Thermo Fisher) and the dsDNA High Sensitivity Assay (Thermo Fisher). DNA quality was checked on an Agilent Bioanalyzer (Agilent, Santa Clara, CA). Libraries for next-generation sequencing were generated using a NEBNext Ultra II gDNA prep kit with NEBNext Multiplex Oligos for Illumina sequencing (New England Biolabs, Ipswich, MA). Prior to library generation, the samples were fragmented using the Covaris S2 system (Covaris, Woburn, MA). The libraries were sequenced in single-end 50 base mode on an Illumina HiSeq 2500 machine (Illumina, San Diego, CA). When more than three samples were prepared, amplified cDNA samples were subjected to PCR analysis to amplify the *H4-GFP* sequence using *H4GFPfor4* and *GFPrev3* primers (see Supplemental Table 2 for sequences). The preparations showing high contrast between GFP-positive and GFP-negative samples were chosen for subsequent RNA-seq analysis.

### LCM

LCM, subsequent RNA extraction and amplification were carried out as previously described (Agusti et al., 2011). Library preparation and RNA sequencing were carried out by BGI Genomics (Shenzhen, China) using the HiSeq 2000 platform (Illumina) in single-end 50 base mode.

### Bioinformatic analyses

The TAIR10 genome sequence was obtained from Ensembl Plants (Arabidopsis_thaliana.TAIR10.28.dna.toplevel.fa) (Arabidopsis Genome, 2000; Bolser et al., 2016). The Araport11 gene annotation file was used (Araport11_GFF3_genes_transposons.201606.gtf) (Cheng et al., 2017), and the chromosome names were changed to fit the TAIR10 genome dataset. The genome index was generated using STAR (v2.5.0a) (Dobin et al., 2013), and FASTQ files were trimmed by Cutadapt (v2.3) (Martin, 2011) using the following options: *-g AAGCAGTGGTATCAACGCAGAGTACGGG -a CCCGTACTCTGCGTTGATACCACTGCTT -g AAGCAGTGGTATCAACGCAGAGTAC -a GTACTCTGCGTTGATACCACTGCTT -b "A 30" -b "T 30 " -n 2 -g AGATCGGAAGAGC -l 50 -m 23 –overlap 5*. Using these settings, oligo sequences used for Smart-seq2 amplification, poly A or T sequences, and Illumina adaptor sequences were removed from the reads. Trimmed files were then mapped to the genome with STAR using the *outFilterMultimapNmax 1 –alignIntronMax 10000 –alignMatesGapMax 10000 outFilterScoreMinOverLread 0.9* options. Intron length limit (10,000 bp) was set based on the characteristics of the Arabidopsis genome (Chang et al., 2017). Please consult Supplemental Table 3 for basic statistics applied to the RNA-seq datasets. For LCM-derived RNA-seq datasets, untrimmed reads were mapped using the same settings. To determine GFP reads, the GFP sequence was indexed by STAR using the *genomeSAindexNbases* 4 option. Subsequently, reads were mapped with STAR using the *–alignIntronMax 1 –alignMatesGapMax 1 –outFilterScoreMinOverLread 0.9* options. Further analysis was carried out in R (v3.5.0). The GTF file was imported using makeTxDbFromGRanges in the GenomicFeatures library (v1.32.3) with the *drop.stop.codons* option (Lawrence et al., 2013). The position of each gene was extracted using the *genes* function, which also included the intron region. Read counts per gene were obtained using summarizeOverlaps in the GenomicAlignments library (v1.16.0) using the *mode = "Union", ignore.strand = TRUE* options (Lawrence et al., 2013). For TPM calculation, the *width* function was used to calculate the length of each gene extracted, which also included intron regions. For the analysis of intron and exon regions, the *intronsByTranscript* and *transcripts* functions were used for genomic range extraction. To analyze intergenic regions, the *gaps* function was used with the extracted gene regions (output of the *genes* function) for genomic range extraction. For comparison with previous datasets, three replicates of the Mock_bdl dataset [deposited in National Center for Biotechnology Information's (NCBI) GEO database (Barrett et al., 2013): GSE98193] were used (Brackmann et al., 2018). Differentially expressed genes were identified using DESeq2 (v1.20.0) (Wald test) with default options to compare GFP-positive and GFP-negative nucleus populations, and the LRT in DESeq2 was used for multiple comparisons (Love et al., 2014). For GFP reads, the inverted beta-binomial test was performed with the *ibb* function (Pham and Jimenez, 2012), using the sum of uniquely mapped reads and multiple mapped reads to the Arabidopsis genome as the total sample count. PCA was carried out using the *plot PCA* function in R using log transformed values obtained from the *rlog* function in the DESeq2 library with the *blind=FALSE* options. Clustering analysis was carried out using the *degPatterns* function in the DEGreport library (v1.16) based on the log transformed values of significantly differentially expressed genes (BH adjustment of *P*-value in LRT <0.01) in the FANS/RNAS-seq-derived datasets. Distances between samples were calculated using the *dist* function in the *stats* library (3.5.0) and visualized in heat maps using *pheatmap*. GO enrichment analysis was carried out using PANTHER (v14.1) through the TAIR10 platform (https://www.arabidopsis.org/tools/go_term_enrichment.jsp) (Mi et al., 2019). Fisher’s exact test was carried out in R. Basic statistics were carried out with Microsoft Excel.

### Determining over-representation of transcription factor binding regions

To determine the over-representation of transcription factor binding regions, 568 genome-wide DAP-Seq profiles for 387 Arabidopsis TFs were downloaded from the Plant Cistrome Database (O'Malley et al., 2016). The background dataset was composed of the 5′-regions (−1500;+1) upstream of the transcription start sites of 19,916 protein-coding genes from the TAIR10 version of the Arabidopsis genome (Lamesch et al., 2012). The foreground dataset was composed of the respective 5′-regions of the SDE genes revealed by FANS/RNA-seq or LCM/RNA-seq experiments. To assess the significance of overlaps between the 5′-regions and the transcription factor binding profiles, we counted the number of foreground/background 5′-regions that contained at least 1 bp overlap with each of the DAP-Seq profiles and applied Fisher’s exact test. A transcription factor was considered to be a potential regulator of the gene set if its binding profile was significantly over-represented in the 5′-regions of the respective genes compared with the rest of the genome under Bonferroni adjusted *P *<* *8.8e−05.

## Accession numbers

Arabidopsis Genome Initiative locus identifiers for each gene are as follows: *NST3* (*AT1G32770*), *VND7* (*AT1G71930*), *PXY* (*AT5G61480*), *SMXL5* (*AT5G57130*), *APL* (*AT1G79430*), *SCR* (*AT3G54220*), *LTP1* (*AT2G38540*), *H4* (*AT5G59690*), *XCP1* (*AT4G35350*), *WOX4* (*AT1G46480*), *MOL1* (*AT5G51350*), *PEAR1* (*AT2G37590*), *PEAR2* (*AT5G02460*), *NAC086* (*AT5G17260*), *PIN3* (*AT1G70940*), *FDH* (*AT2G26250*), *CER6* (*AT1G68530*), *NST1* (*AT2G46770*), *VND6* (*AT5G62380*), *SEOR1* (*AT3G01680*), *VND1* (*AT2G18060*), *VND2* (*AT4G36160*; *NAC076*), *ABI5* (*AT2G36270*), *CAMTA1* (*AT5G09410*), *CAMTA5* (*AT4G16150*), *CBF1* (*AT4G25490*), *CBF2* (*AT4G25470*), *CBF3* (*AT4G25480*; *DREB1A*), *CBF4* (*AT5G51990*), *GBF3* (*AT2G46270*), *RTV1* (*AT1G49480*), *SMB* (*AT1G79580*), *GT2* (*AT1G76890*), *PHV* (*AT1G30490*), *LMI1* (*AT5G03790*), *MYB55* (*AT4G01680*), *MYB83* (*AT3G08500*), *ABF2* (*AT1G45249*), *HY5* (*AT5G11260*), *TINY* (*AT5G25810*), *AREB3* (*AT3G56850*), *ATHB13* (*AT1G69780*), *ERF38* (*AT2G35700*), *ERF34* (*AT2G44940*), *CYP83A1* (*AT4G13770*), *CYP83B1* (*AT4G31500*), *ANAC028* (*AT1G65910*), *ANAC074* (*AT4G28530*), *LHCB3* (*AT5G54270*), *LHCB2.2* (*AT2G05070*), *FMO* (*AT1G12200*), *WRKY28* (*AT4G18170*), *NCED4* (*AT4G19170*), *RBGA6* (*AT4G39260*), *DIN10* (*AT5G20250*), *KIN2* (*AT5G15970*), *COR15B* (*AT2G42530*), *MYB3* (*AT1G22640*), *SEN1* (*AT4G35770*), *HSFA2* (*AT2G26150*), *CYP94B1* (*AT5G63450*), *ANT* (*AT4G37750*), *UCN* (*AT1G51170*), *BG4* (*AT3G13980*), *PIL1* (*AT2G46970*), *DOF2.2* (*AT2G28810*), *ATHMP10* (*AT1G56210*), *LHT7* (*AT4G35180*), *MYB29* (*AT5G07690*), *MIPS3* (*AT5G10170*), *CLE46* (*AT5G59305*), *AGP26* (*AT2G47930*).

Raw data discussed in this publication have been deposited into NCBI’s Gene Expression Omnibus (Barrett et al., 2013) and are accessible through GEO Series accession number GSE142034 at https://www.ncbi.nlm.nih.gov/geo/. In addition, data for genes of interest can be accessed via a website-based tool that allows gene expression profiles to be extracted (https://arabidopsis-stem.cos.uni-heidelberg.de/).

## Supplemental Data

**Supplemental Figure 1.** H4-GFP reporter lines used in this study.

**Supplemental Figure 2.** FANS for GFP-positive and GFP-negative nuclei.

**Supplemental Figure 3.** PCA plot and correlation heatmap of all datasets derived from GFP-positive nuclei of seven different reporter lines.

**Supplemental Figure 4.** Gene expression profiles for epidermis-associated genes.

**Supplemental Figure 5.** Gene expression profiles for each FANS/RNA-seq-derived gene cluster.

**Supplemental Figure 6.** Validation of gene expression patterns determined by FANS/RNA-seq.

**Supplemental Figure 7.** Validation of gene expression patterns determined by FANS/RNA-seq.

**Supplemental Figure 8.** PCA plot and correlation heatmap for FANS-derived and LCM-derived datasets.

**Supplemental Table 1.** Summary of RNA-seq results.

**Supplemental Table 2.** Primers used in this study.

**Supplemental Table 3.** Basic statistics of RNA-seq datasets provided in this study.

**Supplemental Data Set 1.** Differentially expressed genes comparing GFP-positive to GFP-negative nuclei from *NST3_pro_*, *SMXL5_pro_*, and *APL_pro_* lines, respectively.

**Supplemental Data Set 2.** Raw read counts for each FANS/RNA-seq dataset.

**Supplemental Data Set 3.** Raw and normalized read counts for each LCM/RNA-seq dataset.

**Supplemental Data Set 4.** Results of likelihood ratio test (LRT) analyses of FANS/RNA-seq datasets obtained from *NST3_pro_*, *VND7_pro_*, *PXY_pro_*, *SMXL5_pro_*, *APL_pro_*, *SCR_pro_* and *LTP1_pro_* lines.

**Supplemental Data Set 5.** Normalized read counts for each FANS/RNA-seq dataset.

**Supplemental Data Set 6.** Clustering of genes based on their expression patterns among seven tissues analyzed by FANS/RNA-seq.

**Supplemental Data Set 7.** SDE genes comparing the phloem cap and pith with the remaining vascular bundle using the Wald test.

**Supplemental Data Set 8.** GO term enrichment analysis for phloem cap and pith-associated genes.

**Supplemental Data Set 9.** Average values of normalized read counts of SDE genes in each FANS/RNA-seq-derived dataset ranked according to the highest values.

**Supplemental Data Set 10.** SDE genes comparing *NST3_pro_*-positive and *VND7_pro_*-positive nuclei using the Wald test.

**Supplemental Data Set 11.** GO term enrichment analysis for genes predominantly active in *NST3_pro_*-positive versus *VND7_pro_*-positive nuclei, and for genes predominantly active in *VND7_pro_*-positive nuclei versus *NST3_pro_*-positive nuclei.

**Supplemental Data Set 12.** List of genes used for comparison with previously published xylem or phloem-associated expression datasets.

**Supplemental Data Set 13.** GO term enrichment analysis for genes active in *NST3_pro_*, *VND7_pro_*, *PXY_pro_*-positive and *SMXL5_pro_*-, *APL_pro_*-positive domains.

**Supplemental Data Set 14.** Fold enrichment values of significantly over-represented transcription factor binding regions in the upstream regions of genes from FANS/RNA-seq-derived clusters and tissue-specific genes determined by LCM/RNA-seq.

**Supplemental Data Set 15.** List of genes with tissue-specific expression based on FANS/RNA-seq- and LCM/RNA-seq-derived datasets.

**Supplemental Data Set 16.** Sequences of synthesized promoter regions used in this study.

## Supplementary Material

koaa019_Supplementary_Data_Updated

## Notes

T.G. designed the research. D.S., V.J., J.A., V.K., V.V.M. and P.S. performed the research. P.S., D.S., and V.K. generated plant material. V.J. and P.S. developed nucleus isolation and RNA extraction protocols. V.L. and V.V.M. developed the method for enrichment analysis of transcription factor binding regions. D.S., V.J., V.L. and V.V.M. analyzed data. D.S. and T.G. wrote the article.

The author responsible for distribution of materials integral to the findings presented in this article in accordance with the policy described in the Instructions for Authors (www.plantcell.org) is: Thomas Greb (thomas.greb@cos.uni-heidelberg.de).
