# Supplementary material for: Tissue-specific transcriptome profiling of the Arabidopsis inflorescence stem reveals local cellular signatures
Source: Plant Cell. 2020 Dec 2;33(2):200–23. doi: 10.1093/plcell/koaa019 (PMC8136906; doi:10.1093/plcell/koaa019)
Supplement: koaa019_Supplementary_Data_Updated [file koaa019_supplementary_data_updated.zip › tpc.00825.2020-01-Supplemental-Figures-Tables.pdf]

Supplemental Figures and Tables. Shi et al. (2021). Tissue-specific transcriptome profiling of the Arabidopsis inflorescence stem reveals local cellular signatures. Plant Cell.

Supplemental Figures 1-10 Page 1-10
Supplemental Tables 1-3 Page 11-14
listing of Supplemental Data Sets Page 15-17

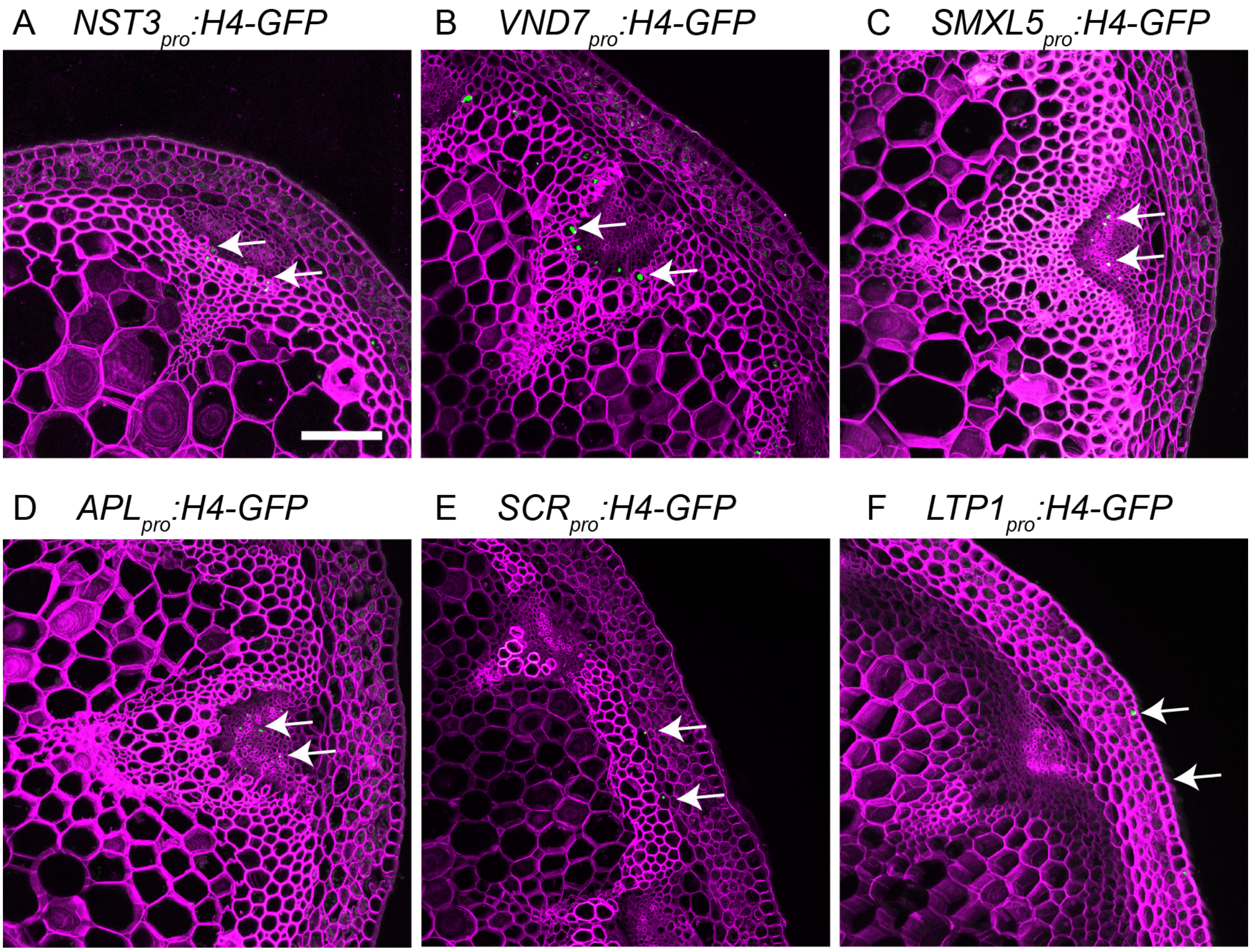

Supplemental Figure 1. H4-GFP reporter lines used in this study (supports Figure 1)

(A-F) Maximum intensity projection of confocal images of cross sections from the second elongated internode. The GFP signal is shown in green and Direct Red 23-stained cell walls are visualized in magenta. Arrows indicate GFP-positive nuclei. Scale bar: 100 µm. Note that only the nuclei in the limited observable depth of the section are detected and visualized.

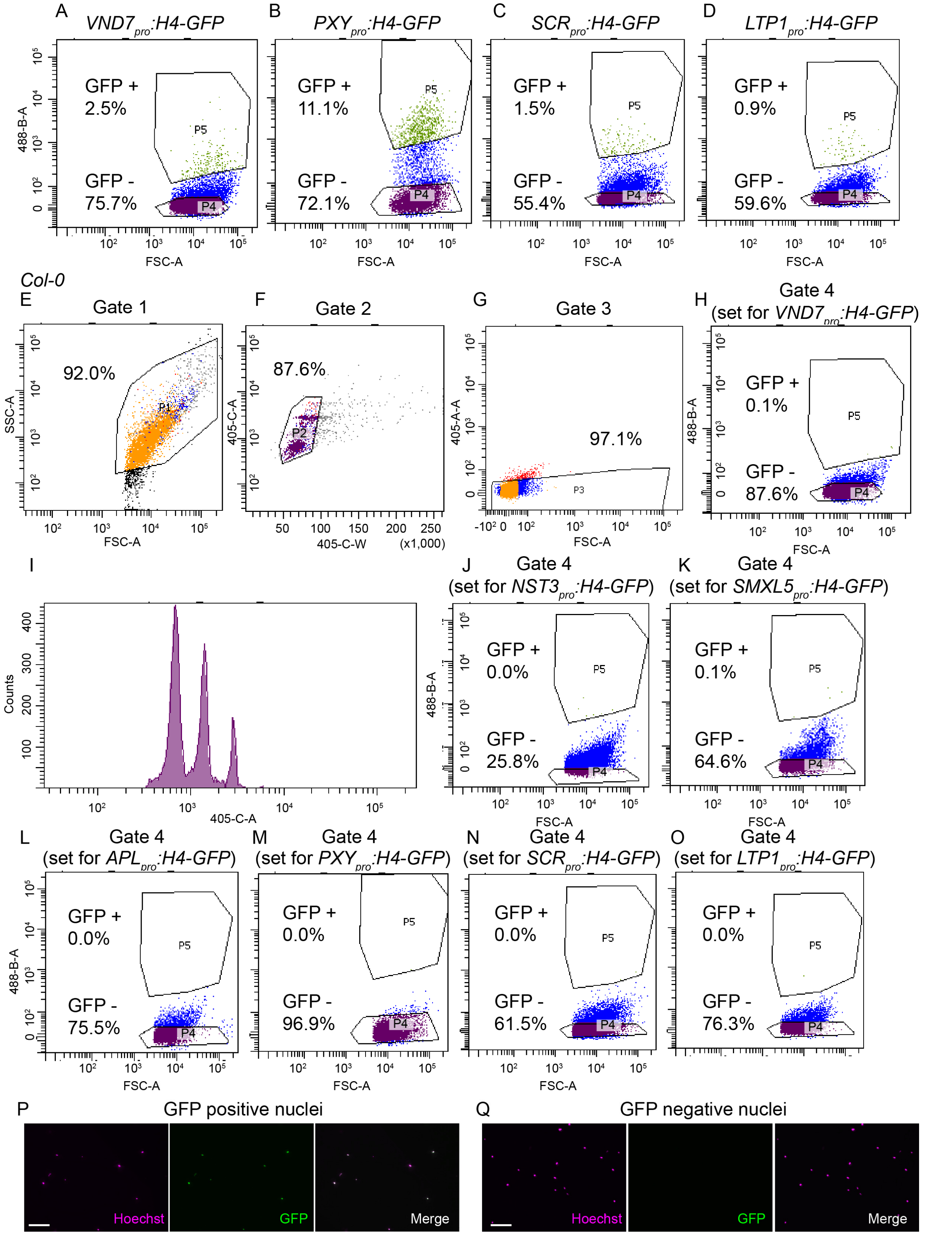

Supplemental Figure 2. FANS for GFP-positive and GFP-negative nuclei (supports Figure 2)

(A-D) Plot of gate settings defining GFP-positive (P5) and GFP-negative nucleus (P4) populations from *VND7*$_{pro}$*:H4-GFP* (A), *PXY*$_{pro}$*:H4-GFP* (B), *SCR*$_{pro}$*:H4-GFP* (C) and *LTP1*$_{pro}$*:H4-GFP* (D)

transgenic plants, respectively. The percentages of each population compared to all events are indicated. X axis (FSC; forward scatter intensity) corresponds to the diameter of the cell, the Y axis (488) indicates levels of GFP fluorescence. (E-H) Additional FANS gate settings. One example from wild type plants without transgene sorted in parallel to samples from *VND7$_{pro}$:H4-GFP* plants is shown. See Nucleus Sorting section in the Methods section for further description. SSC: side scatter (E), 405-C-W: width of fluorescent signal induced by the 405 nm laser, 405-C-A: area of fluorescent signal induced by the 405 nm laser (F), 405-A-A: auto-fluorescence signal detected in the YFP channel excited by the 405 nm laser (G). (H) Same gate settings as used in (A). (I) Histogram showing the distribution of DNA content of nuclei detected by 405 laser excitation after Hoechst staining. (J-O) Wild type plants without transgene sorted with the gate settings used for the respective reporter lines. (P, Q) Microscopic images of sorted GFP-positive and -negative nuclei obtained from the *PXY$_{pro}$:H4-GFP* reporter line. Nuclei were stained with Hoechst (shown in magenta). The GFP signal is shown in green. Scale bar = 100 µm.

Supplemental Data. Shi et al. (2021). Tissue-specific transcriptome profiling of the Arabidopsis inflorescence stem reveals local cellular signatures. Plant Cell.

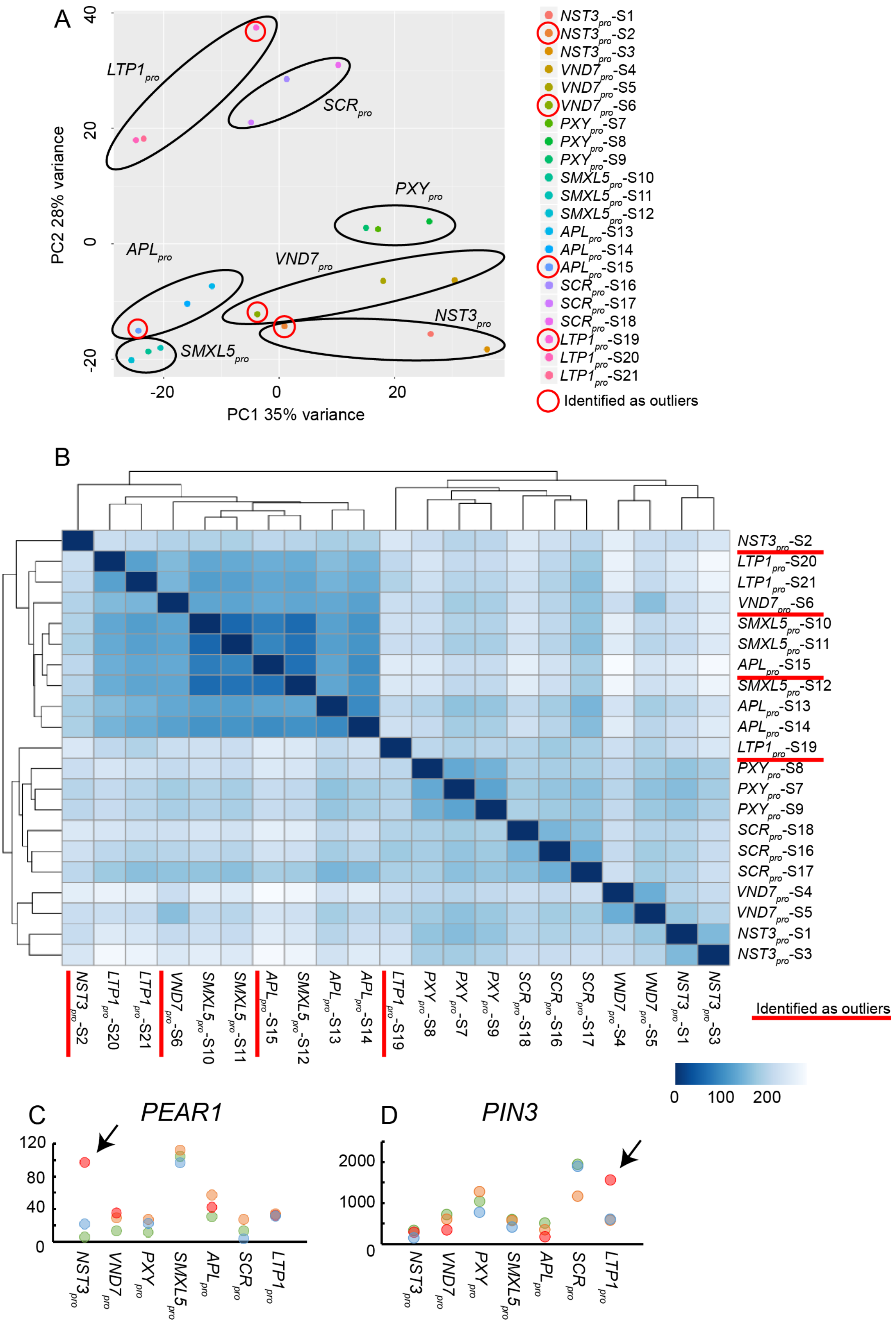

Supplemental Figure 3. PCA plot and correlation heatmap of all datasets derived from GFP-positive nuclei of seven different reporter lines (supports Figure 3).

(A) PCA of log-transformed normalized read counts of each RNA-seq dataset. (B) Heatmap indicating statistical distances between RNA-seq datasets according to the color code. Euclidean

distances [arbitrary unit] between samples were calculated using the *dist* function in R, based on the log2-transformed normalized read counts. Three replicates were obtained from each nucleus population for the seven different promoter lines. Samples marked in red did not cluster together with other replicates of the same nucleus population. (C-D) Normalized read counts for the indicated genes among seven different tissues displayed individually for each replicate including the samples identified as outliers (in red). Arrows indicate the data point from outlier samples that was at odds with known gene expression profiles.

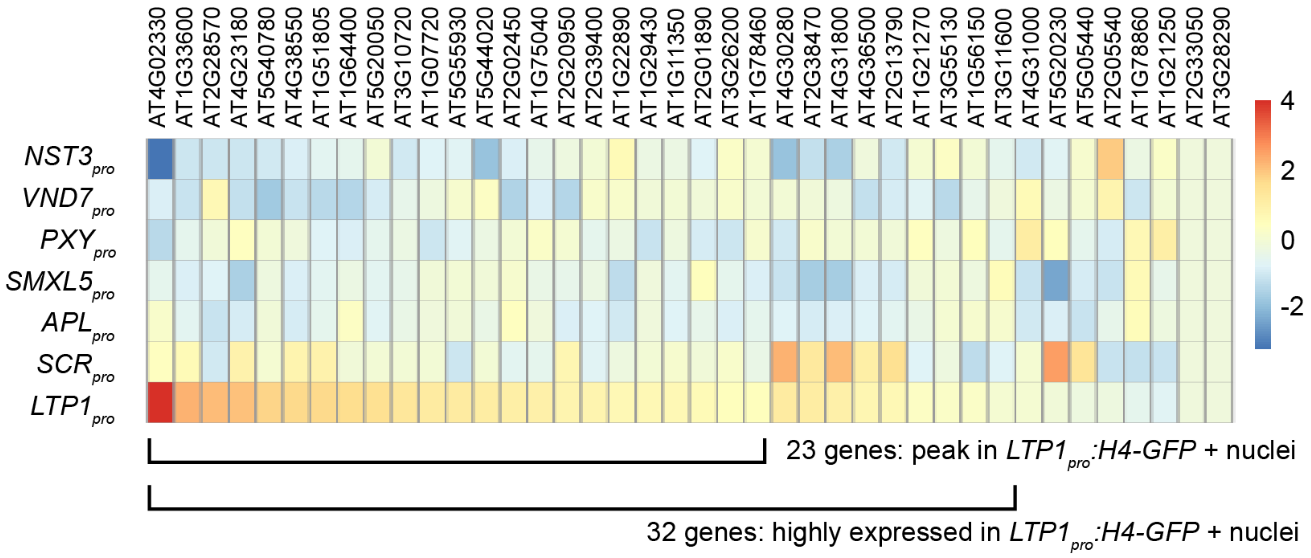

Supplemental Figure 4. Gene expression profiles for epidermis-associated genes (supports Figure 4).

Relative expression heat map of the 40 genes which are most specifically expressed in the epidermis according to Suh et al., 2005 in seven FANS-derived datasets. Relative expression values are color coded and indicate the log2 values of normalized read counts after the average values found in all seven nucleus samples for each gene were subtracted.

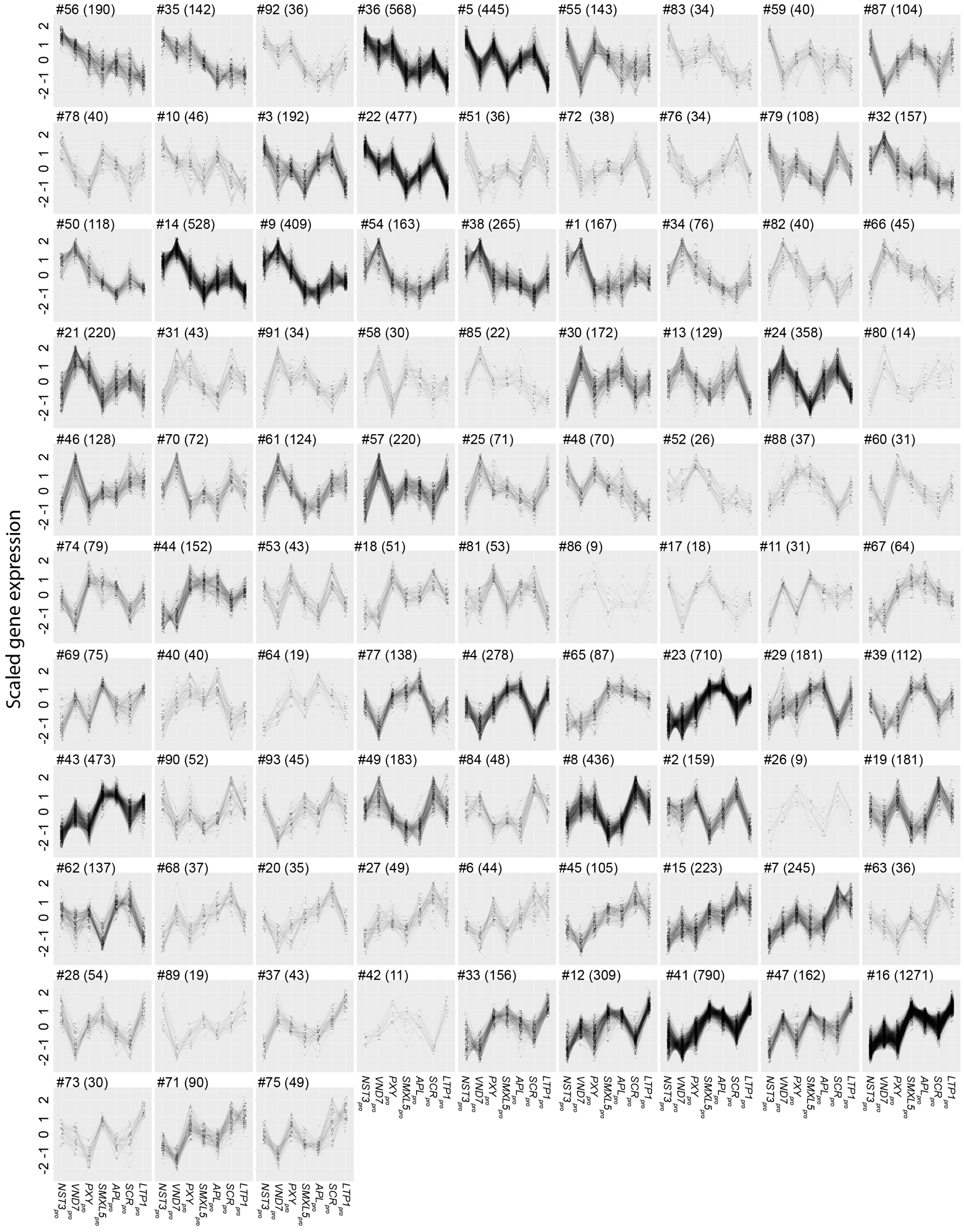

Supplemental Figure 5. Gene expression profiles for each FANS/RNA-seq-derived gene cluster (supports Figure 6).

Scaled relative gene expression values are visualized for each cluster. Normalized read counts were log2-transformed and then the distribution range of each gene among seven nucleus types was again normalized. Labels indicate the cluster ID and the number of genes in each cluster (#Cluster ID (number of genes)). The pattern of expression for each cluster is displayed in the same way as in Figure 6.

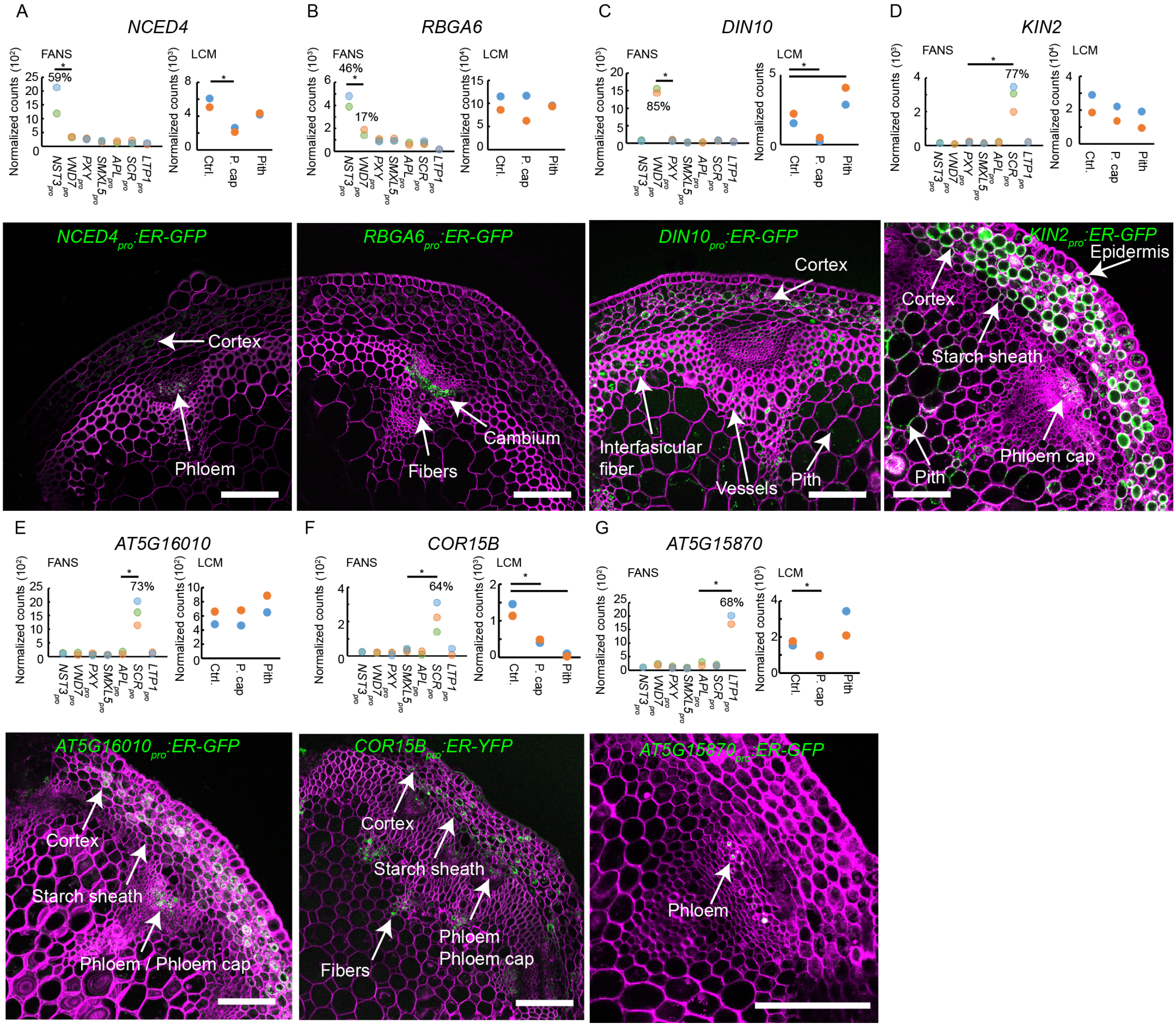

Supplemental Figure 6. Validation of gene expression patterns determined by FANS/RNA-seq (supports Figure 7).

(A-G) Upper panels; normalized read counts found in FANS- and LCM-derived datasets are shown for each indicated gene (*NINE-CIS-EPOXYCAROTENOID DIOXYGENASE 4 (NCED4), RNA-BINDING GLYCINE-RICH PROTEIN A6 (RBGA6), DARK INDUCIBLE 10 (DIN10), KIN2, AT5G16010, COLD-REGUKATED 15B (COR15B), AT5G15870*). Percentages given in the FANS chart (left) indicate the ratio between the average number of normalized read counts in the selected nucleus type and the average number of normalized read counts found in all seven nucleus types. Asterisks indicate significant differences between the highest value and the second highest value in the FANS charts (p<0.01 in Wald test), and significant differences compared to the control tissue in the LCM charts (p<0.01 in Wald test). Bottom panels; confocal microscopy images for each indicated promoter reporter line. The ER-targeted fluorescent signal is shown in green. The Direct Red 23-stained cell wall is visualized in magenta. Scale bars: 100 µm. A single focal plane is shown in each image. At least two independent transgenic plant lines for each promoter reporter were investigated. Detected signals are indicated by white labels.

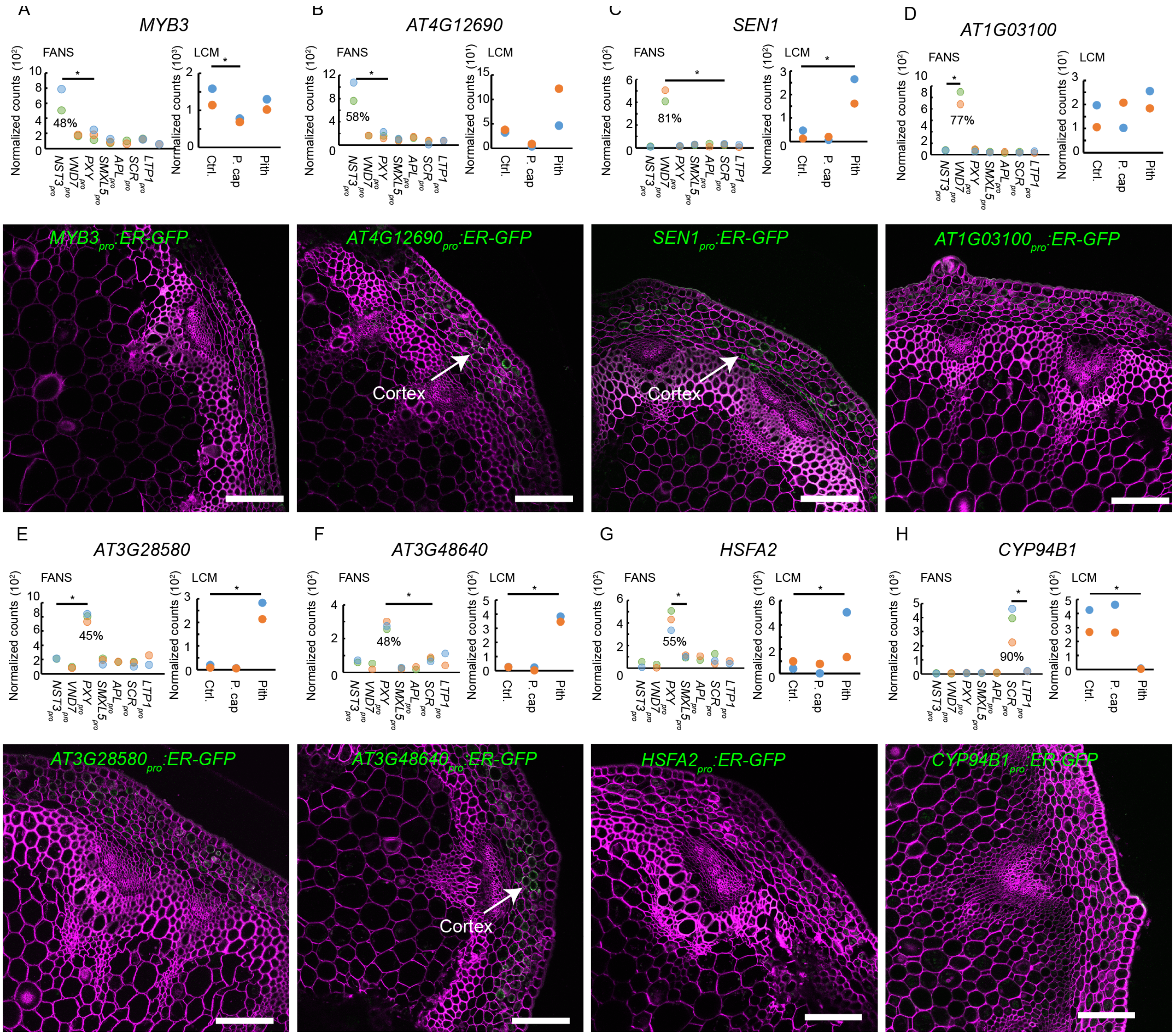

Supplemental Figure 7. Validation of gene expression patterns determined by FANS/RNA-seq (supports Figure 7).

(A-H) Upper panels; normalized read counts found in FANS- and LCM-derived datasets are shown for each indicated gene (*MYB3, AT4G12690, SENESCENCE-ASSOCIATED GENE 1 (SEN1), AT1G03100, AT3G28580, AT3G48640, HEAT SHOCK TRANSCRIPTION FACTOR A2 (HSFA2), CYTOCHROME P450 94B1 (CYP94B1))*. Percentages given in the FANS chart (left) indicate the ratio between the average number of normalized read counts in the selected nucleus type and the average number of normalized read counts found in all seven nucleus types. Asterisks indicate significant difference between the highest value and the second highest value in the FANS charts (p<0.01 in Wald test), and significant differences compared to the control tissue in the LCM charts (p<0.01 in Wald test). Bottom panels; confocal microscopy images for each promoter reporter line. The ER-targeted fluorescent signal is shown in green. The Direct Red 23-stained cell wall is visualized in magenta. Scale bars: 100 µm. A single focal plane is shown in all images. At least two independent transgenic plant lines for each promoter reporter were analyzed. Detected signals are indicated by white labels. Autofluorescence in the green channel was sometimes detected in the cortex, making it difficult to judge whether the signals were derived from the respective reporters.

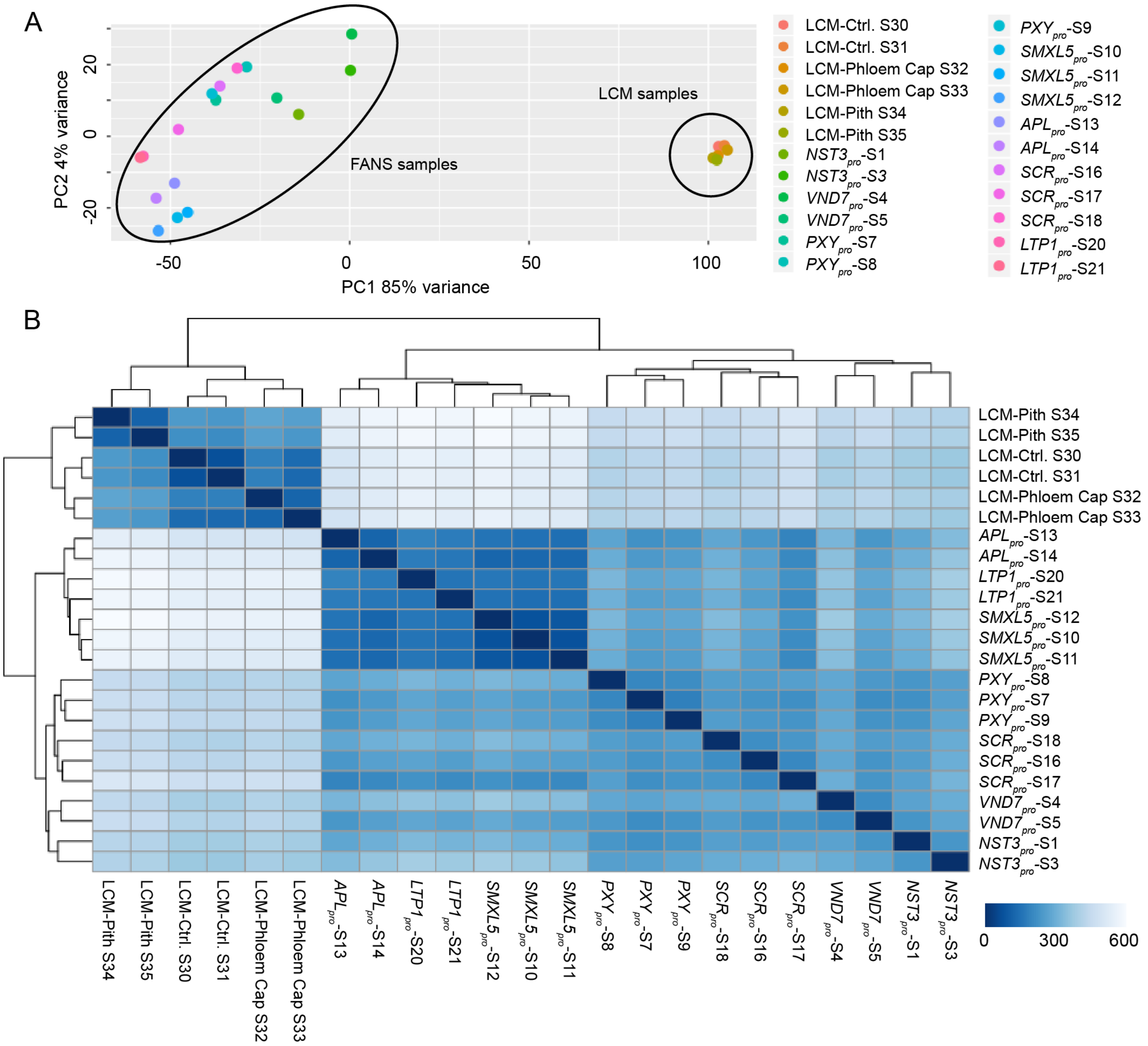

Supplemental Figure 8. PCA plot and correlation heatmap for FANS-derived and LCM-derived datasets (supports Figure 3).

(A) PCA on log-transformed normalized read counts of each RNA-seq dataset. (B) Heatmap indicating statistical distances between RNA-seq dataset according to the color code. Euclidean distance [arbitrary unit] between samples were calculated using the *dist* function in R, based on the log2-transformed normalized read counts.

Supplemental Table 1. Summary of RNA-seq results.

Datasets	Intron reads / exon reads	Intergenic reads / exon reads
**FANS**		
NST3$_{pro}$	14.39%	208%
VND7$_{pro}$	15.67%	219%
PXY$_{pro}$	14.72%	208%
SMXL5$_{pro}$	24.18%	184%
APL$_{pro}$	19.60%	189%
SCR$_{pro}$	16.45%	202%
LTP1$_{pro}$	24.34%	194%
**Whole organ**		
Brackmann *et al.*, 2018	7.68%	203%
**LCM**		
Phloem cap	7.65%	192%
Pith	6.48%	198%
Vascular bundle	7.00%	189%

Summary of RNA-seq results for the different tissues from the second elongated internode analyzed by FANS/RNA-seq, for the whole organ by conventional RNA-seq (Brackmann *et al.*, 2018) and by LCM/RNA-seq. n = 2 or 3 for each dataset.

Supplemental Table 2. Primers used in this study.

Primer name	Sequence (5‘-3‘)	Usage
NST3for3	ACTAGCGGCCGCgattctacacattcacaaagtttactac	NST3 promoter
NST3rev4	GTCGACTGAGATCTAGCCATGGttaacgaagatagc aatatatttttggg	NST3 promoter
NST3for5	CCATGGCTAGATCTCAGTCGACatgattatctatacata cacatacac	NST3 terminator
NST3rev3	ACTAGGTACCacggattcaccatgtgcgtt	NST3 terminator
H4GFPfor4	ACTAACATGTCTtcgggtcgtggaaaggga	Cloning H4GFP into NST3 construct, PCR for H4-GFP
H4GFP-WOX4rev	ACTAGGATCCttatttgtatagttcatccatgc	Cloning H4GFP into NST3 construct
VND7for2	ACTAGCGGCCGCtagcacgttgacgtatgctgag	VND7 promoter
VND7rev2	ACTAGGATCCatC**CATGGccacgatgat**cctataaacgt	VND7 promoter
VND7for3	ACTAGGATCCgaT**CTAGAttaaaaaaaa**acacacttctat atattg	VND7 terminator
VND7rev3	ACTAGGTACCccttctacgtggatcagaagg	VND7 terminator
H4GFPfor5	**ATCATCGTGGCCATG**tcgggtcgtggaaaggga	Cloning H4GFP into VND7 construct
H4GFPrev5	**TTTTTTTTAATCTAG**Agttataaccagtattatttgtatagttc atccatgc	Cloning H4GFP into VND7 construct
H4GFP-APLfor	ACTAACatgtcgggtcgtggaaaggga	Cloning H4GFP into APL construct
H4GFP-APLrev	ACTACTGCAGttatttgtatagttcatccatgc	Cloning H4GFP into APL construct
SCRprom1	ACTAGCGGCCGCcgaccaccaccgtcaacaat	SCR promoter
SCR_Prom_R	ACTAGGATCCAGATGcatggagattgaagggttgttggtc	SCR promoter
SCR_Prom3´_F	ACTACCCGGGcagcttggacgcctcgttcttag	SCR terminator
SCR_Prom3´_R	AGAAATGAATTCagagctccacggtggtgg	SCR terminator
H4GFP-SCRfor	ACTAGGATCCttcgggtcgtggaaaggga	Cloning H4GFP into SCR, LTP1 construct
H4GFP-SCRrev	ACTACCCGGGttatttgtatagttcatccatgc	Cloning H4GFP into SCR, LTP1 construct
LTP1pGreenfor	ACTAGCGGCCGCgaccaaaatgattaac	LTP1 promoter
LTP1pGreenrev	ACTAGGATCCatccatggattgatctcttaggtagt	LTP1 promoter
LTP1prom3'for	ACTACCCGGGtgagctagcaacggtgagatgatg	LTP1 terminator
LTP1prom3'rev	ACTAGGTACCcttttgtggaaatagaaacttggagtta	LTP1 terminator
GFPrev3	tcctctcctgcacgtatccc	PCR for H4-GFP
AT5G20250pro-F	AACAGGTCTCAACCT ttcgtgtaggcgtgtagcactcg	AT5G20250 promoter
AT5G20250pro-R	AACAGGTCTCATGTT tttgtttctctctctctctttggctttc	AT5G20250 promoter
AT1G29520pro-F	AACAGGTCTCAACCT attttctctgttagtagttagttaattacctaatcttagtctc	AT1G29520 promoter
AT1G29520pro-R	AACAGGTCTCATGTT ttcgccttttgaaaaactacgaaagcg	AT1G29520 promoter
AT5G28630pro-F	AACAGGTCTCAACCT aattaagccttatgtttggagcaatgtaaaaggtag	AT5G28630 promoter
AT5G28630pro-R	AACAGGTCTCATGTT cctctttctcttttttattttcttggtgtttttagttc	AT5G28630 promoter
AT5G15970pro-F	AACA GGTCTC A ACCT aaaacgcaaagaaaactttatacagtacatactcttatg	AT5G15970 promoter
AT5G15970pro-R	AACA GGTCTC A TGTT cagatattttttcttgtaaaatcgtttttgatgtgtgttttg	AT5G15970 promoter
AT2G26150pro-F	AACA GGTCTC A ACCT tcgttagaaatgggcttaagtaaaggccc	AT2G26150 promoter
AT2G26150pro-R	AACA GGTCTC A TGTT tttcgttgtttatctcaaatccataagctcagag	AT2G26150 promoter
AT1G12200pro-F	AACA GGTCTC A ACCT	AT1G12200 promoter

	taaatatataatctacggttgtagtgttttgtccaaaag	
AT1G12200pro-R	AACA GGTCTC A TGTT gttaggttatagtgaagttttgtgatctaatgaagaaag	AT1G12200 promoter
AT1G24575pro-F	AACA GGTCTC A ACCT atattatacttgcgcctcaagatgttggc	AT1G24575 promoter
AT1G24575pro-R	AACA GGTCTC A TGTT tcttaaactagctctttcttgtttggatcg	AT1G24575 promoter
AT5G16010pro-F	AACA GGTCTC A ACCT tccaactccacaagttaaaaattttcattaatgc	AT5G16010 promoter
AT5G16010pro-R	AACA GGTCTC A TGTT tttcttttgtttttggtttctcttcggaagaaag	AT5G16010 promoter
AT5G15870pro-F	AACA GGTCTC A ACCT aacactcaaaatggcttttactttctagtatcc	AT5G15870 promoter
AT5G15870pro-R	AACA GGTCTC A TGTT tttgcttagtcaagaggagaagagataacg	AT5G15870 promoter
ANTpro-F	AACA GGTCTC A ACCT ggatccaacctgtccaccataaaaattg	ANT promoter
ANTpro-R	AACA GGTCTC A TGTT ggtttcttttttggtttctgcttctcttc	ANT promoter
AT5G54270for1	**CTGCAGGAATTCGATT**aaatacatatcaaaccccaat	AT5G54270 promoter
AT5G54270rev1	**TAGCTTTAGCTTGAT**tgtctctctcggcttgag	AT5G54270 promoter
AT2G42530for1	**CTGCAGGAATTCGATT**agggtcttacctaatcagt	AT2G42530 promoter
AT2G42530rev1	**TAGCTTTAGCTTGAT**gaggactcttaaagatgtttt	AT2G42530 promoter
AT2G05070for1	**CTGCAGGAATTCGAT**tgtgttccttatattgctttg	AT2G05070 promoter
AT2G05070rev1	**TAGCTTTAGCTTGAT**aatcgtaatctttgtttaactac	AT2G05070 promoter
AT5G59305for1	**CTGCAGGAATTCGAT**ggaaagagctgggatctgc	AT5G59305 promoter
AT5G59305rev1	**TAGCTTTAGCTTGAT**atttcttatgtagagaattag	AT5G59305 promoter
AT2G47930for1	**CTGCAGGAATTCGAT**aatattcgaaaaggcgtccgc	AT2G47930 promoter
AT2G47930rev1	**TAGCTTTAGCTTGAT**ggttactgtgaatgtctctc	AT2G47930 promoter
AT4G35180for1	**CTGCAGGAATTCGAT**tggtgtatatgtgtcaacaag	AT4G35180 promoter
AT4G35180rev1	**TAGCTTTAGCTTGAT**tgtatacaaaataaactttattttc	AT4G35180 promoter
AT5G07690for1	**CTGCAGGAATTCGAT**ggtgtattaagcctatatgag	AT5G07690 promoter
AT5G07690rev1	**TAGCTTTAGCTTGAT**cttgtttttcttcttttttgatatag	AT5G07690 promoter
AT5G10170for1	**CTGCAGGAATTCGAT**gttttgacgttgtgtggaag	AT5G10170 promoter
AT5G10170rev1	**TAGCTTTAGCTTGAT**ttttgcctaaggaagactttg	AT5G10170 promoter
YFP-F1	**ATTCGATATCAAGCT**aaagctaccatggataataagacta a	Cloning ER-EYFP-HDEL into pGREEN0229
YFP-R1	**CGGTATCGATAAGCT**gggttagagttcgtcgtgct	Cloning ER-EYFP-HDEL into pGREEN0229
Clath-F1	**TATCGATACCGTCGA**cctgttttcagataatgtttatccttc	Cloning AT4G24550 terminator into the modified pGREEN0229 vector
Clath-R1	**CCCCCTCGAGGTCGA**atcgttgcacctttatttcttttg	Cloning AT4G24550 terminator into the modified pGREEN0229 vector

ATGC (underline) Sequence for restriction enzyme.
ATGC (bold) Sequence for In-Fusion reaction.
ATGC (uppercase) Sequence added for cloning.
atgc (lowercase) Sequence for annealing.

Supplemental Table 3. Basic statistics of RNA-seq datasets in this study

Region	*ID*	*Raw reads*	*After trim*	*Uniquely mapped*	*Multiply mapped*	*Unmapped*	*Accession Number*
NST3	S1	33,098,137	59.54%	58.13%	23.19%	18.68%	GSM4217858
NST3	S2	26,554,872	46.06%	39.04%	23.10%	37.85%	GSM4217859
NST3	S3	33,622,145	72.47%	71.40%	19.80%	8.81%	GSM4217860
VND7	S4	53,508,021	35.45%	54.56%	36.59%	8.85%	GSM4217867
VND7	S5	28,049,974	40.18%	49.02%	41.24%	9.74%	GSM4217868
VND7	S6	21,234,034	44.60%	60.59%	28.40%	11.01%	GSM4217869
PXY	S7	42,073,803	79.76%	53.76%	29.61%	16.63%	GSM4217870
PXY	S8	52,535,458	82.56%	54.35%	23.82%	21.83%	GSM4217871
PXY	S9	20,301,474	77.91%	53.76%	26.99%	19.25%	GSM4217872
SMXL5	S10	23,176,874	37.69%	57.31%	27.95%	14.75%	GSM4217861
SMXL5	S11	23,424,841	43.37%	56.51%	30.33%	13.16%	GSM4217862
SMXL5	S12	19,248,090	65.06%	66.42%	21.85%	11.73%	GSM4217863
APL	S13	68,143,227	84.51%	55.39%	39.61%	5.00%	GSM4217864
APL	S14	35,571,237	84.91%	60.55%	34.78%	4.67%	GSM4217865
APL	S15	48,232,939	81.70%	70.68%	24.22%	5.09%	GSM4217866
SCR	S16	27,756,903	31.27%	40.23%	40.94%	18.83%	GSM4217873
SCR	S17	12,568,312	53.14%	51.52%	36.81%	11.67%	GSM4217874
SCR	S18	14,885,558	72.22%	57.15%	35.60%	7.25%	GSM4217875
LTP1	S19	64,699,969	22.89%	33.01%	33.66%	33.33%	GSM4217876
LTP1	S20	16,682,896	35.87%	50.14%	26.29%	23.57%	GSM4217877
LTP1	S21	35,493,342	28.41%	42.67%	25.75%	31.58%	GSM4217878
LCMCtl	S30	23,403,325	-	95.51%	1.38%	3.11%	GSM4217888
LCMCtl	S31	24,831,655	-	95.80%	1.25%	2.94%	GSM4217889
Pcap	S32	23,446,707	-	87.50%	1.28%	11.22%	GSM4217890
Pcap	S33	23,226,571	-	93.83%	1.47%	4.70%	GSM4217891
Pith	S34	24,593,314	-	88.65%	1.98%	9.37%	GSM4217892
Pith	S35	23,997,414	-	95.35%	1.47%	3.18%	GSM4217893
GFP negative							
SMXL5	S40	17,901,192	42.59%	58.18%	20.05%	21.77%	GSM4217882
SMXL5	S41	20,436,609	19.77%	37.51%	17.76%	44.72%	GSM4217883
SMXL5	S42	18,359,819	28.85%	48.91%	18.33%	32.76%	GSM4217884
APL	S43	11,087,751	87.91%	59.77%	34.34%	5.88%	GSM4217885
APL	S44	9,220,810	79.36%	47.06%	38.86%	14.08%	GSM4217886
APL	S45	16,792,527	82.77%	58.36%	35.77%	5.86%	GSM4217887
NST3	S46	24,937,918	55.18%	49.67%	26.39%	23.94%	GSM4217879
NST3	S47	15,052,250	44.21%	15.27%	5.13%	79.60%	GSM4217880
NST3	S48	34,229,346	72.57%	64.71%	23.63%	11.66%	GSM4217881

Supplemental Data Set legends

Supplemental Data Set 1. Differentially expressed genes comparing GFP-positive to GFP-negative nuclei from $NST3_{pro}$, $SMXL5_{pro}$ and APL_{pro} lines, respectively.
The output of DESeq2 applying the Wald test is shown. "*baseMean*": mean value of normalized read counts of all the samples used for the comparison. "*log2FoldChange*": log2 values of fold changes of normalized read counts in datasets comparing GFP-positive and GFP-negative nuclei. "*lfcSE*": the standard error estimated for the log2 fold change. "*stat*": Wald statistics. "*pvalue*": Wald test *p*-value. "*padj*": Benjamini-Hochberg adjusted *p*-value.

Supplemental Data Set 2. Raw read counts for each FANS/RNA-seq dataset
Raw read counts for all Arabidopsis genes in all obtained datasets (All_dataset) and in the reduced number of dataset (Reduced_dataset). The name of each dataset corresponds to information given in Figure 3 and Supplemental Figure 3.

Supplemental Data Set 3. Raw and normalized read counts for each LCM/RNA-seq dataset.
Raw and normalized read counts determined by DESeq2 using total counts in each LCM RNA-seq dataset.

Supplemental Data Set 4. Results of likelihood ratio test (LRT) analyses of FANS/RNA-seq datasets obtained from $NST3_{pro}$, $VND7_{pro}$, PXY_{pro}, $SMXL5_{pro}$, APL_{pro}, SCR_{pro} and $LTP1_{pro}$ lines.
The output of DESeq2 using LRT is shown. Please see the legend of Supplemental Dataset 1 for further information. Here, "log2FoldChange" represents the changes between the model with the group of each line and the reduced model. Genes with padj values lower than 0.01 are selected in one table ('SDE genes') and the results of all genes are shown in a second table ('all genes') for comparison.

Supplemental Data Set 5. Normalized read counts for each FANS/RNA-seq dataset
Normalized read counts determined by DESeq2 using total counts in each dataset. SDE genes among seven different tissue types identified by LRT (*padj* < 0.01) are shown in one table ('SDE genes') and the results for all genes are shown in a second table ('all genes') for comparison.

Supplemental Data Set 6. Clustering of genes based on their expression patterns among seven tissues analyzed by FANS/RNA-seq.
SDE genes among seven different tissue types identified by LRT (*padj* < 0.01) were clustered and the cluster ID was assigned to each gene.

Supplemental Data Set 7. SDE genes comparing the phloem cap and pith with the remaining vascular bundle using the Wald test.
The output of DESeq2 using the Wald test is shown. Please see the legend of Supplemental Dataset 1 for further information. Here, "*log2FoldChange*" indicates the log2 value of the fold change of normalized read counts found in phloem cap / pith datasets compared with the remaining vascular bundle. Genes with *padj* > 0.01, *log2FoldChange* > 1 were selected, ranked according to the log2FoldChange and shown for the phloem cap and the pith, respectively.

Supplemental Data Set 8. GO term enrichment analysis for phloem cap and pith-associated genes
The output of the PANTHER Overrepresentation Test using Fisher's Exact test with Bonferroni correction is shown. For a detailed description of each column, please see the 'help' option of PANTHER (http://go.pantherdb.org/tips/tips_overrep.jsp). „Arabidopsis thaliana - REFLIST"

indicates the number of genes registered for each GO term. “phloem cap “or “pith” indicates the number of genes for each GO term in the list of interest. “phloem cap (expected)” indicates the expected number of genes for each GO term. “fold Enrichment” indicates the fold enrichment of the genes observed in the list of interest over the expected value. “over/under” indicates overrepresented or underrepresented terms in the list. Here, only overrepresented terms were selected. “P-value”: *p*-value in Fisher's Exact test with Bonferroni correction corresponds to the false discovery rate. GO terms with *p*-values below 0.05 are shown.

Supplemental Data Set 9. Average values of normalized read counts of SDE genes in each FANS/RNA-seq-derived dataset ranked according to the highest values.
Normalized read counts (Supplemental Data Set 5) of SDE genes identified by LRT (padj<0.01) were averaged for each tissue. The average value and the rank of each tissue are presented. Genes are ranked according to the ratio of the highest value to the second highest value. Wald test (DESeq2 contrast) results between highest and second highest tissue are shown in "p_value (1st and 2nd)" column.

Supplemental Data Set 10. SDE genes comparing *NST3$_{pro}$*-positive and *VND7$_{pro}$*-positive nuclei using the Wald test.
The output of DESeq2 using the Wald test is shown. Please see the legend of Supplemental Dataset 1 for further information. Here, “*log2FoldChange*” indicates the log2 value of fold changes of normalized read counts from *VND7$_{pro}$*-positive nuclei compared to *NST3$_{pro}$*-positive nuclei. 14,063 SDE genes were compared and genes with *padj* > 0.01 and an absolute value of *log2FoldChange* > 1 were selected and ranked according to the log2FoldChange. Thus, genes are ordered from *VND7$_{pro}$* enriched (top) to *NST3$_{pro}$* enriched (bottom).

Supplemental Data Set 11. GO term enrichment analysis for genes predominantly active in *NST3$_{pro}$*-positive versus *VND7$_{pro}$*-positive nuclei, and for genes predominantly active in *VND7$_{pro}$*-positive nuclei versus *NST3$_{pro}$*-positive nuclei.
The output of the PANTHER Overrepresentation Test using the Fisher's Exact test with Bonferroni correction is shown. GO terms with a *p*-value below 0.05 are presented. Please see the legend of Supplemental Data Set 8 for further information.

Supplemental Data Set 12. List of genes used for comparison with previously published xylem or phloem-associated expression datasets.
All genes analyzed by microarrays and the genes enriched in APL, SUC2, S32, S17, S4, S18, JO121 samples were obtained from Brady et al., 2006. S4, S18, JO121-associated genes were combined as *xylem* genes and SUC2, S32, APL, S17-associated genes were combined as *phloem* genes in the processed data section. All the genes expressed in *Populus tremula* stems annotated to Arabidopsis genes were obtained from Sundell et al., 2017 and duplicates were removed. Genes clustered as b1 (phloem-specific expression pattern), and g1, g2 (lignified xylem-specific expression pattern) are listed with *P. tremula* gene codes and the annotated Arabidopsis gene codes without duplicates in the processed data section. “*NST3$_{pro}$, VND7$_{pro}$, PXY$_{pro}$*-enriched genes” and “*SMXL5$_{pro}$, APL$_{pro}$*-enriched genes” were obtained from SDE gene clusters determined by the FANS/RNA-seq datasets (Supplemental Dataset 6). The associated cluster ID for each gene is shown in the *cluster ID* column. These two lists were further filtered by *Microarray genes* or *Expressed genes in P. tremula annotated to Arabidopsis genes (de-duplicated)*, and the filtered genes are shown in the *NST3$_{pro}$, VND7$_{pro}$, PXY$_{pro}$* and *SMXL5$_{pro}$, APL$_{pro}$* columns for each comparison.

Supplemental Data Set 13. GO term enrichment analysis for genes active in *NST3$_{pro}$, VND7$_{pro}$, PXY$_{pro}$*-positive and *SMXL5$_{pro}$*-, *APL$_{pro}$*-positive domains.
The output of PANTHER Overrepresentation Tests using the Fisher's Exact test with Bonferroni correction is shown. GO terms with *p*-value below 0.05 are presented. Please see the legend of

Supplemental Data Set 8 for further information. Gene lists used for this analysis can be found in Supplemental Data Set 12.

Supplemental Data Set 14. Fold enrichment values of significantly over-represented transcription factor binding regions in the upstream regions of genes from FANS/RNA-seq-derived clusters and tissue-specific genes determined by LCM/RNA-seq.
Shown are fold enrichment values of each transcription factor binding region in promoters from FANS/RNA-seq cluster genes and in the phloem cap and pith-associated genes. AGI_TF indicates the Arabidopsis Genome Initiative (AGI) codes for each associated transcription factor. DAP_ID indicates the binding region ID determined by DNA affinity purification sequencing (O'Malley et al., 2016). Only significantly over-represented regions are shown (p < 8.8e-05 (Bonferroni adjusted threshold of 0.05) in Fisher's exact test).

Supplemental Data Set 15. List of genes with tissue-specific expression based on FANS/RNA-seq- and LCM/RNA-seq-derived datasets.
For FANS/RNA-seq-derived datasets, SDE genes were first ranked according to the domain with the highest expression value (Supplemental Data Set 9), then genes with at least two-fold higher expression in the domain with the highest value in comparison to any other domain and whose expression value in the domain with the highest value was significantly different from the domain with the second highest value (p < 0.01, Wald test) were selected. For LCM-derived datasets, SDE genes compared to the control tissue (vascular bundle) (Supplemental Data Set 7) were selected (BH adjustment of p value in Wald test < 0.01, fold change > 2). Finally, duplicates within these lists were removed.

Supplemental Data Set 16. Sequences of synthesized promoter regions used in this study.
The promoter sequence used for each reporter is shown. Underlined sequences were added for conducting the Greengate reaction.
