# Supplementary material for: Tissue-specific transcriptome profiling of the Arabidopsis inflorescence stem reveals local cellular signatures
Source: Plant Cell. 2020 Dec 2;33(2):200–23. doi: 10.1093/plcell/koaa019 (PMC8136906; doi:10.1093/plcell/koaa019)
Supplement: koaa019_Supplementary_Data_Updated [file tpc.00825.2020-19-PeerReviewReport.pdf › tpc.00825.2020-19-PeerReviewReport.pdf]

Tissue-specific transcriptome profiling of the Arabidopsis inflorescence stem reveals local cellular signatures

Dongbo Shi, Virginie Jouannet, Javier Agustí, Verena Kaul, Victor Levitsky, Pablo Sanchez, Victoria V Mironova, Thomas Greb

Corresponding author: Thomas Greb thomas.greb@cos.uni-heidelberg.de.

Review timeline:

**TPC2020-00106-LSB**	Submission received:	Feb. 13, 2020
	1st Decision:	March 17, 2020 *manuscript declined*
**TPC2020-00825-LSB**	Submission received:	Oct. 5, 2020
	1st Decision:	Oct. 28, 2020 *revision requested*
**TPC2020-00825-LSB1**	1st Revision received:	Nov. 2, 2020
	2nd Decision:	Nov. 2, 2020 *acceptance pending, sent to science editor*
	Final acceptance:	Nov. 12, 2020

REPORT: (The report shows the major requests for revision and author responses. Minor comments for revision and miscellaneous correspondence are not included. The original format may not be reflected in this compilation, but the reviewer comments and author responses are not edited, except to correct minor typographical or spelling errors that could be a source of ambiguity.)

TPC2020-00106-LSB 1st Editorial decision – *declined* Feb. 13, 2020

All reviewers found your work interesting but requested further analysis of the data, including better descriptions and cross referencing to other data sets. In addition, Reviewer 3 requested additional experimental validation of the data. As the reviewer points out, based on the table shown in Figure 7G, the patterns of only 3 of the 9 genes tested are perfectly validated, and all of these centre around phloem patterns. Therefore, in the post-review consultation, we agreed to ask you for more extensive validation. This will need to include additional experimental data, such as in situ hybridizations or more transgenic plants carrying promoter fusions to markers. However, it might also include more extensive cross-referencing with other data sets, and perhaps some reassessment of the predicted patterns in Figure 7G. For example, AT5G20250 is predicted to be expressed only in vessels in Figure 7G, but there are clearly reads in the fibers (NST3) sample in Figure 7A. Therefore, perhaps the predicted patterns in Figure 7G are over-simplified. We also request that you consider all of the comments of the reviewers and provide a detailed response to the reviewers along with any resubmission. Based on the extensive nature of the revisions requested, including experimentation, we decided to decline this version, but we would be willing to consider a revised version that convincingly answers the points raised by the reviewers.

--- Reviewer comments:

[Reviewer comments shown below along with author responses]

TPC2020-00825-LSB Submission received Oct. 5, 2020

Reviewer comments on previously declined manuscript and **author responses:**

Reviewer #1:

Shi et al. well described and represented tissue-specific gene expression patterns in stem tissues. FANS with several distinct tissue-specific markers and LCM analysis newly revealed tissue-specific transcriptome datasets. In the manuscript, the authors confirmed and validated their expression patterns by promoter reporters. In addition, these expression data are easy to access for investigating the expression profile of a gene-of-interest. In this respect, I think that this manuscript is well written and is suitable for a Large-Scale Biology Article. However, I feel that the obtained datasets are not fully discussed in comparison with other datasets. Here I put few major and minor comments below.

Point 1. Detection of outliers: The authors removed 4 datasets as outliers based on PCA and heatmap clustering. Usually, it is hard to find out which are outliers among 3 independent biological samples. I am wondering how the authors define the outliers in this experiment. Otherwise, the authors should include all of these obtained data for further analyses.

RESPONSE: We agree with the concerns raised by the reviewer. We assumed that each biological replicate from the same reporter line should cluster together and not cluster with samples from other reporter lines in the PCA. This criterion was chosen for defining 'outliers'. As a result, we eliminated some samples that showed unexpected patterns. As the purpose of this study is to provide a tissue-specific gene expression atlas, which can recapitulate known gene expression profiles, we prefer to use the reduced dataset for downstream analysis. To make this point clear, we added a paragraph to the text (page 11), provide all datasets including those classified as 'outliers' and provide examples of genes whose expression is at odds with described expression pattern in Supplemental Figure 3.

Point 2. Comparison with the previous transcriptome datasets: In Fig. 8, it seemed that the isolated SDE genes are not well-overlapping with the root FACS data (Brady et al., 2006), which simply reminds us of the methodological difference between with nuclei and with protoplasts. The problem is that PXYpro>SMXL5pro genes contain not only vascular-related genes but also non-vascular-related genes. Here I recommend using the clustering gene sets shown in Fig. 6 for the Venn-diagram analysis. PXYpro-enriched clusters should over-represent xylem-related genes rather than phloem-related genes. The case of GO enrichment analysis is the same. Such vascular-focused analyses would be a better verification.

RESPONSE: We thank the reviewer for this suggestion and, as a response, we tested this idea. PXYpro-enriched clusters (48, 52, 60, 74, 53, 18, 81, 86) contain 284 genes, which were also identified as being differentially expressed between the different root tissues in Brady et al. However, only 11 genes of those are xylem-associated genes according to Brady et al., which is not significantly enriched. We assume that genes associated with mature xylem cells are not necessarily the most highly expressed in the PXY domain and may be expressed in other domains like the differentiated xylem domain.

Therefore, we combined all the NST3pro, VND7pro and PXYpro-related clusters (49 clusters, 5,724 genes in total), containing 719 xylem-associated genes (significantly enriched) according to Brady et al., and 176 phloem-associated genes (neither significantly enriched nor depleted). We also combined SMXL5pro and APLpro-related clusters (13 clusters, 1,449 genes in total) and found that this list contained 146 phloem-associated genes according to Brady et al. (significantly enriched) and 58 xylem-associated genes (significantly depleted). Since the enrichment and the number of overlapping genes are higher than the previous version using PXYpro>SMXL5pro or SMXL5pro>PXYpro comparisons, we modified our manuscript accordingly. The text was modified and is now found on page 26-28 and results are displayed in Figure 10.

Point 3. Another point is overlapping genes between PXYpro and SMXL5pro. Because the previous paper by the authors' group (Shi et al., 2019 Development) showed that the overlapping region of PXY and SMXL5 expression is the cambial stem cells, the authors should discuss this aspect with the both expressed cluster in more detail. Also, ANT is the good marker for vascular stem cells. The authors should present ANT expression patterns in Fig. 4.

RESPONSE: We definitely appreciate this recommendation. As the reviewers (#1, #3) suggested, we analyzed cluster 88 containing genes whose expression was high in both PXYpro and SMXL5pro domains in more detail. ANT was indeed included in this cluster, and we also confirmed its expression by analysing a novel promoter reporter line. Furthermore, we selected five genes from this cluster and found that promoter reporters of all of these genes were active in the cambium domain including cambium stem cells. The text explaining and discussing this aspect is now found on page 23-24, and data are presented in Figure 8.

Point 4. Transcriptome analysis using LCM: In the Abstract and Discussion, the authors emphasized the utility of LCM datasets. I agree that LCM analysis is a good way to uncover the tissue-specific expression from an alternative approach, but LCM datasets were only shown and discussed in Fig. 5. Here I recommend a comparison between LCM and FANS datasets to validate both transcriptome data. Besides, integrating these two different datasets will be useful for more accurate gene clustering. The authors should effectively utilize the LCM datasets.

RESPONSE: Thanks to the reviewers (#1, #3) for this suggestion. We tried to incorporate LCM datasets into our clustering analysis, but as FANS-derived and LCM-derived datasets were very different from each other, we could not obtain meaningful clustering results. When doing so, most of expressed genes were classified as differentially

expressed genes because of the large value differences between the different dataset types (FANS or LCM). Unfortunately, we also could not develop a normalization method to include LCM datasets into our clustering analysis, so far. We added a discussion of this point in the revised manuscript on page 35.

However, although we could not directly compare FANS-derived and LCM-derived datasets due to the above-mentioned reason, we added the expression values from LCM-derived datasets to all the figures describing promoter reporter line analyses (Figure 7-9, Supplemental Figure 6-7). We also added promoter reporter line analyses to validate LCM-based predictions of gene expression patterns in the stem (Figure 9), and provide lists of tissue-associated genes (Supplemental Dataset 15) to further integrate the LCM-based analyses into our manuscript.

Reviewer #2:

The authors profiled the transcriptomes of nine different tissues of Arabidopsis inflorescence stems. They also confirmed in-vivo localisation of a subset of the newly identified tissue-specific genes and performed in-silico analysis of gene regulatory networks. The manuscript provides a new valuable data set for the plant science community, the data and methodology are well described, and a webpage has been generated to easily access the data.

Point 1. The authors provide a nice web page to navigate their data set. A link to the website is provided in the Discussion (line 387). The possibility to easily access to the data represents a main strength of the paper, therefore I strongly recommend to add the link to the website at in the abstract.

RESPONSE: Thanks to the reviewer for this suggestion. We modified our abstract accordingly.

Point 2. For the data represented in Fig. 3, 5C and S3, it is not clear how the correlation between the samples was calculated. In the legend of Fig. 3, 5C and S3, the authors must specify the type of correlation used: was that a Pearson correlation? For a correlation, values between -1 and 1 are expected, whereas in the figures, the color scale is from 0 to 200.

RESPONSE: In the heatmap, Euclidean distance [arbitrary unit] between samples was calculated by the dist function in R and shown according to the color code, based on log2-transformed normalized read counts. Thus, we did not perform Pearson correlation or any type of correlation. We now make this point clear in the respective figure legends.

Point 3. The authors wrote "genes characterizing developing phloem cells (SMXL5pro) were, by large, distinct from genes characterizing developing xylem cells" (Line 260). "by large" implies a quantification of the similarity among the samples and this is not provided in the figure. I suggest to add a dendrogram to the heatmap to provide information on the hierarchical distribution of the samples. This will highlight which samples are more similar and more distinct.

RESPONSE: We added a dendrogram to Figure 3 according to the suggestion.

Point 4. Fig.7 must be substantially revised. First, the panels in the figure need to be reorganised because Fig. 7G is mentioned as first in the text (Line 267), and therefore it must be Fig.7A. Second, the authors detect expression of AT5G20250, AT5G15970 and AT5G16010 in the pith and the phloem cap. Were these genes predicted to be expressed in these domains based on the LCM data? Because the authors profiled the transcriptomes of pith and phloem cap tissues, the prediction of expression in these two tissues must be added to the table in Fig. 7G. Finally, in the table, AT1G12200 is marked as detected in the phloem. However, in Fig S6F, I do not see GFP signal in the phloem, while it appears more in the cortex. For AT1G25575, in Fig S6H there is GFP also in the cortex.

RESPONSE: According to the reviewer's suggestions, we substantially revised our promoter reporter line analysis part. In particular, we substantially extended the number of analysed reporters, including analyses for three promoter reporter lines for phloem cap-associated genes and promoter reporter analyses for two pith-associated genes; the results are shown in Figure 9. The related text is found on page 23-25.

For the last point, we occasionally detected autofluorescence signal in the cortex tissue, which carries a characteristic pattern compared to an ER-localized reporter signal. We added this notion into the legend of Figure 7 and Supplemental Figure 7.

Reviewer #3:

In the manuscript submitted by Shi et al., the authors bypassed the challenges of preparing protoplasts for tissue-specific transcriptome profiling from several difficult tissues in stems by employing fluorescence activated nucleus

sorting (FANS) and Laser capture microdissection (LCM). They successfully obtained RNAseq data representing distinct tissues and validated in silico most known marker genes for each tissue type. They aimed to identify tissue-specific genes and enriched transcription factor binding sites for each tissue based on various bioinformatic analyses. They also attempted to validate the predicted genes by analyzing the transcriptional reporter lines for 9 candidate genes. The manuscript provides useful and relative high-resolution transcript profiling data that is relevant for large group of researchers studying vascular tissues or stems. However, the current data are not sufficient, and especially the experimental validation is minimal.

Point 1. The goal of this manuscript is to identify tissue-specific molecular signatures for distinct tissues of Arabidopsis fluorescent stems. To achieve this, the authors performed SDE genes analysis in combination with hierarchical clustering to detect genes that are enriched in certain tissue(s) marked by the seven marker genes used in FANs (Supp. Datasets 4-7, Fig. 6). However, it was not clear how SDE was defined, and thus, how the tissue-enriched genes were selected. Related to that, statistical significance (e.g., padj value) for the fold change (1st /2nd highest value) is missing in Supp. Dataset 7, and therefore, it is impossible to define the tissue-specific genes.

RESPONSE: In Supp. Datasets 4-6, Fig.6, the SDE genes were selected by applying the LRT test, which tested whether these genes have differential expression among seven types of tissues. Although these genes are not necessarily to be highly expressed in only one tissue type, we think the list is quite useful for cluster analysis and for the readers. We made this point clear in the revision of the main text (page 16).

As also mentioned by reviewer #2, tissue-specific gene expression lists are of interest for a broad readership. In the revised manuscript, we added statistical significance values to the comparison with the 1st and 2nd highest expressing tissue in Supp. Dataset 9, and also added a new dataset (Supp. Dataset 15) showing tissue-specific genes that were selected by statistical significance and fold change.

Point 2. To verify the predicted SDE genes in certain tissue types, the authors selected 9 genes and validated their expression by cloning their promoter reporter lines. Based on comparison between predicted and detected signals, the authors concluded "the predictive power of our FANS/RNA-Seq-based transcriptional profiles was high." In my opinion, this statement is not convincing. First, the authors didn't indicate the criteria for selecting genes for validation. There seemed tissue bias on selecting the candidates, for example, no genes for cambium, phloem cap or pith were picked, whereas several genes from phloem sides were analysed. More important, among the 9 genes, only 3 could be largely validated. The authors attributed the results to various reasons. I would suggest the authors validate more genes covering all tissues or utilise a different method (e.g., RNA in situ hybridisation) to confirm the expression patterns of the predicted SDE genes in order to demonstrate the power of their pipeline and the reliability of the data. Especially, the less known cell types, such as pith, phloem cap, cambium or fibers, require experimental verification, and thus, would increase the novelty of their manuscript.

RESPONSE: According to the reviewer's suggestion, we added 25 promoter reporter line analyses to our revised manuscript to explore in more detail the predictive power of our RNA-seq datasets. Thereby, we could identify new genes expressed in the pith, phloem cap, cambium stem cells and fibers. These data are now displayed in Figure 7-9. In total, we found that the expression pattern of 19 out of 34 genes analysed was as expected, with 8 reporter lines showing no signal in the target tissue and 7 reporter lines showing different patterns to the estimated one. Based on these results, we concluded that our FANS/RNA-Seq-based transcriptional profiles can be used for estimating unknown gene expression patterns. Here, single tissue specifically expressed genes in FANS dataset (Figure 7, Supplemental Dataset 9), SDE genes in cluster #88, which are expressed in cambium region (Figure 8, Supplemental Dataset 6) and phloem cap or pith associated genes derived from LCM dataset (Figure 9, Supplemental Dataset 7) For details, please refer to "Tissue-specific mRNA profiling identifies cell type-associated gene promoters" (page 18-25) in our revised manuscript. We agree that RNA in situ hybridisation would be a powerful tool. However, due to the technical difficulties in detecting gene expression by RNA-ISH in differentiated cells with large vacuoles and little cytoplasm in cross sections like in the pith or the xylem, we focused on reporter promoter analysis in our study.

Point 3. This is not a first spatial transcript profiling study in stems. There are several published data sets carried out in Poplar stems (serial cryofractioning followed by transcript profiling). This approach should be mentioned in the Introduction. Also, the authors should take advantage of these region/cell type-specific profiling data to validate their own FANS and laser capture data.

RESPONSE: We thank the reviewer for this suggestion. We now reference transcriptional profiling studies in aspen in

the Introduction (page 5). We also added comparisons of our datasets (NST3pro, VND7pro, PXYpro-enriched, and SMXL5pro, APLpro-enriched clusters) with AspWood datasets (phloem expressing genes and lignified xylem expressing genes) generated in *Populus tremula* (Sundell et al., 2017). The new text describing this comparison is found on page 27-29, and data are displayed in Figure 10.

Point 4. Vascular tissues (xylem and phloem) and their meristem (vascular cambium) are very central tissues in the stem. The authors were able to identify genes that are enriched in xylem and phloem, however, they did not report cambium-enriched genes. The authors should discuss this aspect. It is reported by the same group that *PXY* and *SMXL* are expressed in the proximal and distal cambium, respectively, with overlapping manner in cambium. Therefore, the authors should be able to predict cambium-specific genes by comparing the data from *PXYpro* and *SMXLpro* profiling.

RESPONSE: Many thanks for this comment. As the reviewers (#1, #3) suggested, we analyzed cluster 88 containing genes whose expression was high in both *PXYpro* and *SMXL5pro* domains in more detail (see also above). *ANT* was indeed included in this cluster, and we also confirmed its expression by analysing a novel promoter reporter line. Furthermore, we selected five genes from this cluster and found that promoter reporters of all of these genes were active in the cambium domain, including cambium stem cells. The text explaining and discussing this aspect is now found on page 23-24 and data are presented in Figure 8.

Point 5. Can the authors integrate/compare the LCM-based data with FANS based data? How the phloem cap and pith different from other stem tissues? The current analysis only confirmed previously reported genes in phloem cap and pith. Some experimental gene expression data is needed to validate the data (as mentioned earlier in point 2).

RESPONSE: Thanks to reviewers (#1, #3) for this suggestion. We tried to incorporate LCM datasets into our clustering analysis, but as FANS-derived and LCM-derived datasets were very different from each other, we could not obtain meaningful clustering results. When doing so, almost all expressed genes were classified as differentially expressed genes because of the large value differences between the different dataset types (FANS or LCM). Unfortunately, we also could not develop a normalization method to include LCM datasets into our clustering analysis, so far. We added a discussion of this point in the revised manuscript on page 35.

However, although we could not directly compare FANS-derived and LCM-derived datasets due to the above mentioned reason, we added the expression values from LCM-derived datasets to all the figures describing promoter reporter line analyses (Figure 7-9, Supplemental Figure 6-7). We also added promoter reporter line analyses to validate LCM-based predictions of gene expression patterns in the stem (Figure 9), and provide lists of tissue-associated genes (Supplemental Dataset 15) to further integrate the LCM-based analyses into our manuscript.

TPC2020-00825-LSB 1st Editorial decision – *revision requested*	Oct. 28, 2020

We received comments from two reviewers of the previous version of your manuscript. As you will see, both found the manuscript much improved by the changes that you made and the additional data. Reviewer 2 now requests a small textual change. Reviewer 3 makes suggestions for improving the description of some of the datasets and requests that the magnification of some of the images is increased. Please consider all of these suggestions and edit the manuscript accordingly. We look forward to receiving your revised text and figures.

-- Reviewer comments:

[Provided below along with author responses]

TPC2020-00825-LSB1 1st Revision received	Nov. 2, 2020

Reviewer comments on previous submission and **author responses**:

<u>Reviewer #2:</u>
The Authors strongly improved the manuscript, addressing all the comments raised by the reviewers.
The revised version of the manuscript provides a more extensive validation of the data, and the LCM data are better integrated in the analysis. The authors now provide data of in vivo expression profile for 34 promoter-GFP fusion constructs. However, is to be noted that the percentage of tested case that confirm the expression pattern obtained with FACS and LCM is still limited: Of the 23 promoter reporters tested, only eight behaved as predicted in the tissue-specific expression analysis by FACS. As the author mention, the low number of positive hits could be due to missing

regulatory elements in the construct used or also to low level of expression of certain promoter-fusion that does not allow detection by confocal microscopy.

Point 1. In line 357, the authors state: "In summary, we confirmed a strong association of reporter activities with the predicted tissues in 56 % (19/34) of the cases selected from the different datasets". In my opinion, 56% is not high enough to claim a "strong association"; the sentence should be reformulated.

RESPONSE: We modified the text accordingly.

Reviewer #3:

The added data in the manuscript by Shi et al., including the cambium-enriched genes, verification of various tissue-enriched genes in stem and comparisons with other datasets, have improved the manuscript. I am satisfied with most aspects. I have only a few remaining comments.

Point 1. If I understand correctly, to present tissue-enriched genes, the authors reported high-ranked genes in each FANS-derived dataset (supplementary dataset 9) and selected candidate genes based on this for the promoter testing (Figure 7). On the other hand, the authors also ran hierarchical clustering to identify a number of clusters of genes that are enriched in each tissue (Figure 6 and Supplementary dataset 6). Based on the clustering data, the authors defined, although not clearly indicated, the tissue-associated (line 388, 389 et al) or enriched genes (line 419 et al) and used such gene lists for comparisons with previously published data in roots or tree stems (Figure 10 and Supplementary dataset 12). Could the authors explain why these two types of data were used? And what is important for readers, which dataset is the most useful for identifying tissue-enriched genes? Some of the tissue-specific gene lists in Supplemental Dataset 15 are very short compared to the clustering data - why is that?

RESPONSE: We thank the reviewer for pointing this out. Tissue-type associated gene lists in FANS-derived data (dataset 9) were generated simply according to their ranks. Upon requests from reviewers, it is now combined with the LCM dataset in dataset 15. We applied this method as it is simple and intuitive to obtain a tissue-associated gene list. However, for example, since gene expression in $SMXL5_{pro}$ and APL_{pro} domains are generally shared, there were not many genes specifically expressed in only $SMXL5_{pro}$ or APL_{pro} domains, resulting in the very short gene list in Supplemental Dataset 15. For this reason, we also did clustering analysis, which can capture more complex expression patterns where readers can easily extract genes with any expression pattern. We think the readers will benefit from all these datasets. We added more explanation to the Discussion section to guide the readers.

Point 2. I appreciate that the authors included 25 more promoter reporter lines to demonstrate the predictive power of the dataset. In some cases when the expression domain is narrow, it is difficult to recognize the signals in current confocal images (Figure 7D-7F, 8B, 8E et al) and distinguish phloem and phloem caps (Figure 9A-9C). In these cases, could the authors insert magnified images (e.g. inset)?

RESPONSE: We added magnified images as insets in these figures. We also believe that our original high-resolution figure files would enable the readers to identify the signals.

Point 3. Figure 10C and 10D, what does the depleted genes mean? For example, there are more overlapped depleted genes than enriched genes when comparing "SDEs enriched in PXYpro, NSTpro, VND7pro" (xylem genes) with "phloem expressed genes" in roots or Populus stem. Fig. 10 is not so easy to understand...

RESPONSE: We changed our wording in the manuscript and Figure 10 to make it more comprehensible.

TPC2020-00825-LSB1 2nd Editorial decision – *acceptance pending* Nov. 2, 2020

We are pleased to inform you that your paper entitled "Tissue-specific transcriptome profiling of the Arabidopsis thaliana inflorescence stem reveals local cellular signatures" has been accepted for publication in The Plant Cell, pending a final minor editorial review by journal staff. At this stage, your manuscript will be evaluated by a Science Editor with respect to its presentation of scientific content, compliance with journal policies, and presentation for a broad readership.

Final acceptance from Science Editor Nov. 12, 2020
